# Geminin overexpression prevents the completion of topoisomerase IIα chromosome decatenation, leading to aneuploidy in human mammary epithelial cells

**DOI:** 10.1186/bcr2884

**Published:** 2011-05-19

**Authors:** Lauren Gardner, Rohit Malik, Yoshiko Shimizu, Nicole Mullins, Wael M ElShamy

**Affiliations:** 1 Cancer Institute and Department of Biochemistry, University of Mississippi Medical Center, 2500 N. State Street, G362, Jackson, MS 39216, USA

## Abstract

**Introduction:**

The nuclear enzyme topoisomerase IIα (TopoIIα) is able to cleave DNA in a reversible manner, making it a valuable target for agents such as etoposide that trap the enzyme in a covalent bond with the 5′ DNA end to which it cleaves. This prevents DNA religation and triggers cell death in cancer cells. However, development of resistance to these agents limits their therapeutic use. In this study, we examined the therapeutic targeting of geminin for improving the therapeutic potential of TopoIIα agents.

**Methods:**

Human mammary epithelial (HME) cells and several breast cancer cell lines were used in this study. Geminin, TopoIIα and cell division cycle 7 (Cdc7) silencing were done using specific small interfering RNA. Transit or stable inducible overexpression of these proteins and casein kinase Iε (CKIε) were also used, as well as several pharmacological inhibitors that target TopoIIα, Cdc7 or CKIε. We manipulated HME cells that expressed H2B-GFP, or did not, to detect chromosome bridges. Immunoprecipitation and direct Western blot analysis were used to detect interactions between these proteins and their total expression, respectively, whereas interactions on chromosomal arms were detected using a trapped in agarose DNA immunostaining assay. TopoIIα phosphorylation by Cdc7 or CKIε was done using an *in vitro *kinase assay. The TopoGen decatenation kit was used to measure TopoIIα decatenation activity. Finally, a comet assay and metaphase chromosome spread were used to detect chromosome breakage and changes in chromosome condensation or numbers, respectively.

**Results:**

We found that geminin and TopoIIα interact primarily in G_2_/M/early G_1 _cells on chromosomes, that geminin recruits TopoIIα to chromosomal decatenation sites or *vice versa *and that geminin silencing in HME cells triggers the formation of chromosome bridges by suppressing TopoIIα access to chromosomal arms. CKIε kinase phosphorylates and positively regulates TopoIIα chromosome localization and function. CKIε kinase overexpression or Cdc7 kinase silencing, which we show phosphorylates TopoIIα *in vitro*, restored DNA decatenation and chromosome segregation in geminin-silenced cells before triggering cell death. *In vivo*, at normal concentration, geminin recruits the deSUMOylating sentrin-specific proteases SENP1 and SENP2 enzymes to deSUMOylate chromosome-bound TopoIIα and promote its release from chromosomes following completion of DNA decatenation. In cells overexpressing geminin, premature departure of TopoIIα from chromosomes is thought to be due to the fact that geminin recruits more of these deSUMOylating enzymes, or recruits them earlier, to bound TopoIIα. This triggers premature release of TopoIIα from chromosomes, which we propose induces aneuploidy in HME cells, since chromosome breakage generated through this mechanism were not sensed and/or repaired and the cell cycle was not arrested. Expression of mitosis-inducing proteins such as cyclin A and cell division kinase 1 was also increased in these cells because of the overexpression of geminin.

**Conclusions:**

TopoIIα recruitment and its chromosome decatenation function require a normal level of geminin. Geminin silencing induces a cytokinetic checkpoint in which Cdc7 phosphorylates TopoIIα and inhibits its chromosomal recruitment and decatenation and/or segregation function. Geminin overexpression prematurely deSUMOylates TopoIIα, triggering its premature departure from chromosomes and leading to chromosomal abnormalities and the formation of aneuploid, drug-resistant cancer cells. On the basis of our findings, we propose that therapeutic targeting of geminin is essential for improving the therapeutic potential of TopoIIα agents.

## Introduction

In eukaryotes, the initiation of DNA replication involves the formation and activation of the prereplication complex (pre-RC) at the origins of replication (ORIs). The pre-RCs are formed by the sequential binding of the origin recognition complex (ORC1 to ORC6), cell division cycle 6 (Cdc6), Cdt1 and minichromosome maintenance (MCM2 to MCM7) proteins to DNA [1]. Since loading of the MCM complex onto ORIs is the rate-limiting step in DNA replication, its recruitment to ORIs is inhibited by geminin, the only known endogenous inhibitor of DNA replication. Thus, geminin level and/or activity seem to control the assembly of pre-RCs at ORIs and to determine whether the origins are licensed [2-7].

Geminin, a multifunctional small protein (about 30 kDa), was first identified in a screen for proteins degraded during mitosis using *Xenopus *egg extracts [8-11]. Since then, however, roles for geminin during mitosis have been described [12-20], arguing against its mitotic degradation, at least in mammalian cells. More precisely, geminin silencing in human mammary epithelial (HME) cells [12] or mouse embryos [14], while showing minimal effect on S-phase progression, completely blocked the progress through mitosis [12]. The HME mitosis-arrested cells (due to geminin silencing) showed increased expression and activity of cyclin B1, checkpoint protein 1 (Chk1), and Cdc7 [12]. Surprisingly, only Cdc7 cosilencing triggered apoptosis in geminin-silenced cells [12], implying that Cdc7 is the kinase that maintains the cytokinetic checkpoint induced by geminin silencing in HME cells [12].

The Cdc7-Dbf4 complex is essential for ORI firing and maintenance of replication forks [21-26]. Cdc7 inactivation in cancer cell lines causes growth arrest and cell death, while only arresting growth in normal cells [27]. Although the mechanism of cancer-specific cell death is not yet defined, it is possible that insufficient levels of Cdc7 during cell division may result in stalled and incomplete replication forks, induction of genetic instability and cell death by entering aberrant mitosis in a p53-independent manner [28-30].

Topoisomerases (Topo) are multifunctional enzymes that resolve topological chromosomal complexities, such as knots, tangles and catenanes, arising during DNA metabolism [31]. Yeasts and *Drosophila *cells contain a single type II Topo (TopoII), whereas mammalian cells possess two TopoII isoforms, α and β (TopoIIα and TopoIIβ). Both enzymes can facilitate transcription and replication of chromatin templates [32,33]. However, only TopoIIα is absolutely required for DNA decatenation and chromatid separation during anaphase [34,35]. During DNA decatenation, TopoIIα dimer binds a DNA helix and hydrolyzes adenosine triphosphate (ATP) to introduce a transient double-stranded break (DSB) through which it passes the other entangled intact helix. Then the DNA DSB is religated, and TopoIIα dissociates from the DNA [32-35]. Furthermore, TopoIIα binding to chromosomes and its decatenation activity are modified by phosphorylation and SUMOylation [36-39]. For example, casein kinase Iε (CKIε) phosphorylates TopoIIα on serine 1106 (S1106) in G_2_/M cells and induces the TopoIIα chromosome localization and decatenation function as well as sensitivity to TopoIIα-targeting drugs [40-42]. Moreover, the complex RAN binding protein 2/ubiquitin-conjugating enzyme 9 (RanBP2/Ubc9) SUMOylates TopoIIα and triggers its chromosome translocation and decatenation activity [39].

TopoIIα's ability to cleave DNA in a reversible manner makes it an ideal target for agents such as doxorubicin and etoposide, which poison the enzyme via the trapping of the transient reaction intermediate composed of TopoIIα bound covalently to the 5′ end of the cleaved DNA strands (cleavable complexes), preventing religation of DNA [43]. It thus induces DNA damage, genomic instability and cell death [42,44]. However, development of resistance to these agents limits their therapeutic use [45]. Therefore, an understanding of the mechanisms that lead to the development of this resistance is essential to the improvement of the therapeutic potential of these agents.

In the present study, we show that geminin silencing induces chromosome bridge formation by inhibiting TopoIIα chromosome localization and function. Cdc7 cosilencing or CKIε overexpression in geminin-silenced cells restored TopoIIα chromosomal localization and prevented the formation of chromosome bridges. This finding suggests that CKIε is a positive regulator and Cdc7 is a negative regulator of TopoIIα chromosomal localization and function. However, these cells underwent apoptotic cell death, suggesting that they were unprepared to enter G_1_. Moreover, geminin and TopoIIα interact on chromosomes in G_2_/M/early G_1 _cells, and geminin overexpression prematurely releases TopoIIα from chromosomes, in part by enhancing TopoIIα deSUMOylation on chromosomes. Geminin overexpression also inhibits DNA decatenation before the religation step, leading to linearization of model entangled DNA *in vitro *and chromosome breakage and aneuploidy *in vivo*. These effects were accompanied by decreased cytotoxicity to TopoIIα inhibitors. Importantly, Cdc7 co-overexpression corrected both defects. These data represent a potential mechanism for TopoIIα drug resistance and suggest that inhibiting the activity of geminin and TopoIIα, CKIε and/or Cdc7 can be more beneficial for breast cancer patients with aggressive, drug-resistant disease.

## Materials and methods

### Cell culture and drug treatments

All cells were cultivated in RPMI 1640 Medium (Gibco, Grand Island, NY, USA) containing 10% fetal bovine serum (FBS) (Gemini Laboratories, Inc, West Sacramento, CA, USA) at 37°C in a 10% CO_2_-containing atmosphere unless otherwise mentioned, except HME cells that were maintained in growth factor-supplemented Dulbecco's modified Eagle's medium/Ham's F-12 mammary epithelium basal medium (MEBM) (Clonetics/Cambrex, Walkersville, MD, USA). For fluorescence-activated cell sorting (FACS) analysis, treated cells were fixed in 100% ethanol, stained with 2.5 μg/mL propidium iodide (PI) (Sigma, St. Louis, MO, USA), supplemented with RNase A and incubated at 37°C for one or two hours. A HME cell line that carries a pBOS-H2B plasmid (Clontech Laboratories, Mountain View, CA, USA) was generated by standard plasmid transfection, and clones were selected with blastocidin (Sigma). Etoposide, doxorubicin and IC261 were obtained from Sigma, ICRF187 and PHA767491 were purchased from Tocris Bioscienc (Ellisville, Missouri, USA) and ICRF193 was obtained from Funakoshi (Tokyo, Japan). All drugs were dissolved in dimethyl sulfoxide (DMSO).

### Geminin cloning and bacterial expression

The protocol described by Nakuci *et al*. [12] was used. In brief, geminin full-length cDNA was amplified from IMR90 total RNA using the following primers cut with *Bam*HI/*Sal*I and ligated to the glutathione *S*-transferase (GST) vector pGEX-4T2 cut with the same enzymes: forward 5′-CGGGATCCATGAATCCCAGTATGAAGCAGAAACAAGAA-3′ and reverse 5′-ACGCGTCGACTCATATACATGGCTTTGCATCCGTA-3′. The GST-fused geminin was expressed in competent bacteria One Shot BL21 Star (DE3)pLysS (Invitrogen, Carlsbad, CA, USA), induced with isopropyl-β-D-thiogalactoside and purified on Glutathione Sepharose™ 4B beads (GE healthcare, Piscataway, NJ, USA) and eluted from the beads using 10 mM glutathione in 50 mM Tris HCl, pH 8.0. Using a similar strategy, geminin full-length cDNA was also ligated to the retrovirus plasmid Rev-Tre (Clontech), and the retrovirus was prepared and used to infect the HME cell line expressing the inducer pTet-ON (Clontech). Geminin clones were generated by appropriate selection.

### Antibodies

Mouse anti-geminin antibody (Ab) generation was described earlier [12]. We used mAbs α-cyclin A1, anti-cyclin E, anti-cyclin B1 and anti-CKIε (610445; BD Transduction Laboratories (San Jose, CA, USA); mAb α-actin (Ab-1; Oncogene Science, Cambridge, MA, USA); rAb α-Cdk2 (Pharmingen, San Jose, CA, USA); rAb α-pChk1 (Cell Signaling Technology, Danvers, MA, USA); mAb α-cdc7 (MS-1888-P; NeoMarkers, Fremont, CA, USA); mAb α-cdc2 (B-6), anti-Chk1 (G-4), rAb α-Sp1 (H-225), anti-geminin (FL- 209) and gAb α-lamin B (C-20, sc-6216) (Santa Cruz Biotechnology, Santa Cruz, CA, USA); rAb α-H2B (ab18977) and mAb α-TopoIIα (ab52934) (Abcam, Cambridge, MA, USA); and rAb α-CKII (Millipore, Danvers, Massachusetts:, USA).

### Cell synchronization and small interfering RNA transfection

HME cells were incubated in growth factor-free medium for 72 hours to produce cells in G_0_/G_1 _phase (> 95%) [12]. G_0_/G_1 _cells were then released from arrest in medium containing growth factors, and 16 hours (S phase), 22 hours (G_2_/M phase) or 24 hours (M/G_1 _phase) later cells were collected and analyzed. HME cell synchronization and transfection were performed as described by ElShamy and Livingston [46]. In brief, cells were transfected (0 hours) in serum-free medium with a relevant double-stranded RNA interference reagent by a standard method using oligofectamine. At 24 hours, the medium was changed, and growth factor-containing MEBM (Clonetics/Cambrex) was added. Small interfering RNA (siRNA) used were siGem: TGAGCTGTCCGCAGGCTTT, scrambled siGem: TGATTTGTCCGCAGCTGGC, siCdc7: TTTGTGAACACCTTTCCTGTT and siTopoIIα: sc-36695 (Santa Cruz Biotechnology). The silenced luciferase (siLuc) and silenced green fluorescence protein (siGFP) used were from previously published data.

### Kinase assay

Cells were collected by trypsinization and washed twice with phosphate-buffered saline (PBS). Whole cell extract was prepared by rocking cells in EBC buffer (50 mM Tris HCl, pH 8.0, 0.5% Nonidet P-40 (NP-40) and 120 mM NaCl) at 4°C for 30 minutes and centrifuged at high speed for 15 minutes. Protein A beads were added to the supernatant along with antibodies (anti-Sp1, anti-CKIε or anti-Cdc7) for 2 to 2.5 hours. Beads were washed once with NETN lysis buffer containing 250 mM NaCl, twice with NETN containing 150 mM NaCl and once with kinase buffer (50 mM Tris HCl, pH 7.5, 10 mM MgCl_2 _and 1 mM dithiothreitol (DTT), added fresh). Twenty microliters of kinase buffer were added to the beads, along with 7 μL of ATP mix (5 μL from 40 μM cold ATP + 2 μL of radioactive ATP (20 μCi)) and 10 or 100 ng of purified TopoIIα (TopoGen, Columbus, OH, USA). The reaction was rocked at room temperature for 45 minutes and then stopped by adding 20 μL of sodium dodecyl sulfate (SDS) loading buffer and boiling for 10 minutes.

### Cell sonication, chromatin purification, Western blot analysis and immunoprecipitation

The protocols used by Nakuci *et al*. [12] and ElShamy and Livingston [46] were used to isolate total extracts by sonication and chromatin preparations. Briefly, cells at about 75% confluence were washed several times with PBS and trypsinized. After being washed, 1 × 10^7 ^cells were resuspended in 1 mL of Buffer A (110 mM KC_2_H_3_O_2_, 15 mM NaC_2_H_3_O_2_, 2 mM MgC_2_H_3_O_2_, 0.5 mM ethylene glycol tetraacetic acid and 20 mM 4-(2-hydroxyethyl)-1-piperazineethanesulfonic acid (HEPES), pH 7.3). Next, we added 2 mM DTT and 50 μg/mL digotinin to the cell suspension. The cells were agitated at 4°C for 10 minutes. Nuclei were pelleted by centrifugation in a swinging bucket rotor at 1,500 × *g *for 10 minutes. They were resuspended in hypotonic Buffer B (1 mM HEPES, pH 7.5, and 0.5 mM ethylenediaminetetraacetic acid (EDTA) supplemented with 0.5% NP-40). Typically, a nuclear pellet of about 50 μL was resuspended in 0.5 mL of Buffer B. The nuclear suspension was then agitated at 4°C for 15 minutes and layered on top of a 10-mL sucrose cushion (100 mM sucrose, 0.5 mM Tris HCl, pH 8.5), then centrifuged at 3,500 × *g *for 15 minutes at 4°C. The chromatin pallet was suspended in 0.25 mM EDTA, pH 8.0, and sonicated three times for 10 seconds each, each time using a Fisher Scientific Model 100 Sonic Dimembrator (Fisher Scientific, Pittsburgh, PA, USA). After sonication, the chromatin suspension was centrifuged twice at high speed for 10 minutes at 4°C, and the supernatants were retained. This chromatin extract was first precleared by agitation for 2 hours at 4°C in the presence of 50 μg of protein A/G Sepharose beads, followed by pelleting of the beads. The supernatant protein concentration was measured, and 500 μg of chromatin protein were routinely immunoprecipitated using 1 or 2 μg of Ab and 50 μL of protein A/G Sepharose beads in a total volume of 1 mL of NETN buffer (in which the NaCl concentration was preset at 250 to 500 mM). In some experiments, the deSUMOylation inhibitor *N*-ethylmaleimide (10 nM) was added to sonicates.

### Immunofluorescence analysis

Cells were seeded on slide chambers (LabTek, Rochester, NY, USA) at 25% confluence 24 hours prior to processing. Cells were fixed with 4% paraformaldehyde for 10 minutes at room temperature, permeabilized in Triton X-100 buffer (0.5% Triton X-100 in 20 mM HEPES, pH 7.4, 50 mM NaCl, 3 mM Mg_2_Cl and 300 mM sucrose containing 0.5% bovine serum albumin (BSA)) for 10 minutes at 4°C. Cells were then incubated for 30 minutes with 5% normal mouse or rabbit serum (MS or RS) in PBS and then incubated for 30 minutes at 37°C with primary antibody. Cells were then incubated with appropriate fluorescein isothiocyanate- or rhodamine-conjugated secondary antibodies diluted 1:5,000 to 1:10,000 in 5% MS or RS in PBS for 30 minutes at 37°C. Coverslips were then mounted in anti-fade solution (Vector Laboratories, Burlingame, CA, USA) supplemented with 4′6-diamidino-2-phenylindole (DAPI).

### Comet assay

A neutral comet assay was performed to detect DSBs. After induction of geminin by 2 μg/mL doxycycline (Dox) for 72 hours, cells embedded in agarose were lysed and subjected to electrophoresis as described previously [47]. Individual cells stained with 0.5 μg/mL DAPI were viewed using an ultraviolet (UV) light fluorescence microscope (Olympus, San-Diego, CA, USA). Quantification was achieved by analyzing *x *randomly selected comets per slide with Komet 5.5 software (Kinetic Imaging, Bath, UK) using the variable Olive Tail Moment (with results measured in arbitrary units, defined as the product of the percentage of DNA in the tail multiplied by the tail length).

### Trapped in agarose immunostaining assay

A 50-μL quantity of cell suspension medium warmed to 37°C was mixed with an equal volume of agarose solution (2% (wt/vol) in PBS, SeaPrep Agarose ultralow gelling; FMC BioProducts, Rockland, ME, USA), which had been melted and kept at 37°C. The mixture was immediately spread evenly across a microscope slide and quickly gelled by placing the slides onto a cold surface (0°C). Slides were lysed for 15 minutes at 20°C in a buffer containing 1% (wt/vol) SDS, 80 mM phosphate buffer, pH 6.8, 10 mM EDTA and a protease inhibitor mixture (final concentrations 2 μg/mL pepstatin A, 2 μg/mL leupeptin, 1 mM phenylmethylsulfonyl fluoride, 1 mM benzamidine and 1 mM DTT). Slides were next immersed in 1 M NaCl supplemented with the protease inhibitor mixture for 30 minutes at 20°C, then washed by immersion three times (5 minutes per wash) in PBS. Immunofluorescence was performed according to a protocol described earlier [12]. Slides were counterstained with Hoechst 33258 blue (10 μM in PBS; Sigma) for 5 minutes before application of coverslips that were secured with a sealant.

### TopoGen decatenation assay

TopoIIα enzymatic activity was assayed by measuring the decatenation of *kinetoplast (k)-DNA *(TopoGen). A standard assay carried out in a total volume of 20 μL included 50 mM Tris HCl, pH 7.9, 88 mM KCl, 10 mM MgCl_2_, 0.5 mM EDTA, 10 mM ATP, 10 mM DTT, 100 μg/mL BSA and 300 ng of *k-DNA*. The reaction mixture containing TopoIIα immunoprecipitated from control or siRNA-treated cells was incubated at 37°C, and the reaction was stopped by the addition of 5 μL of stop solution (5% SDS, 25% Ficoll and 0.05% bromophenol blue). The samples were resolved by electrophoresis at 115 V using a 1% agarose gel in Tris-acetate-EDTA buffer with 0.5 μg/mL ethidium bromide and photographed under UV illumination.

### Relaxation of *pBR322 *plasmid negative supercoiled assay

The reactions were carried out by incubating 150 ng of supercoiled *pBR322 *plasmid DNA at 37°C in 15 μL of reaction buffer (10 mM Tris HCl, pH 7.4, 5 mM MgCl_2_, 100 mM KCl and 0.5 mM ATP) and were initiated by the addition of TopoII (1 or 2 U; TopoGen) and different concentrations of GST-geminin as indicated or 100 ng of GST alone (control). Reactions were stopped by the addition of 5 μL of loading buffer, and samples were electrophoresed in 1% agarose gel in Tris-Borate-EDTA (TBE) buffer (pH 8.0) at 10 V/cm for 4 hours. The gel was then stained with TBE containing ethidium bromide (0.5 μg/mL) for 10 minutes, washed extensively and photographed.

### Metaphase spread

Colcemid (100 ng/mL) was added directly to the culture dish and swirled and incubated for 1 hour. Following incubation, cells were trypsinized and washed with PBS. After the cells were washed, all excess PBS was removed and cells were gently resuspended in the residual PBS. KCl (0.075 M) was added slowly dropwise to a quantity of 10 mL to the cells resuspended in PBS. The reaction was incubated at 37°C (in a water bath) for 5 to 10 minutes. The reaction was centrifuged at 900 rpm for 5 minutes, followed by removal of as much KCl as possible, and then the cells were gently resuspended in the residual PBS. Five milliliters of freshly prepared fixative (3:1 methanol to acetic acid) were then added dropwise to the cells and carefully mixed. After centrifugation of the reaction at 900 rpm for 5 minutes and removal of the fixative solution, the whole step was repeated with 2 mL of fixative. Finally, after removing all but 300 μL of the fixative, the cell mixture was dropped from about 18 inches onto an angled, humidified microscope slide and air-dried for at least 10 minutes. Next, PI or Giemsa stain was used to stain the chromosome spread.

### MTT and activated caspases 3 and 7 assays

MTT and activated caspases 3 and 7 assays were done using the CellTiter 96 AQ_ueous _One Solution Cell Proliferation Assay (G3580; Promega, Madison, WI, USA) or the Caspase-Glo 3/7 Assay (G8091; Promega), respectively, according to the manufacturer's instructions. Measurements were obtained using optical density at 490 nm. Each experiment was done in eight samples, and the whole experiment was repeated three times.

### Cell cycle analysis

Cell cycle analysis was carried out by flow cytometery after PI staining using a standard protocol.

### Statistical analysis

Comparisons of treatment outcomes were tested for statistically significant differences using Student's *t*-test for paired data. Statistical significance was assumed at *P ≤ 0.05, **P ≤ 0.01 and ***P ≤ 0.001.

## Results

### Geminin silencing promotes formation of chromosome bridges in HME cells

**Figure 1:**
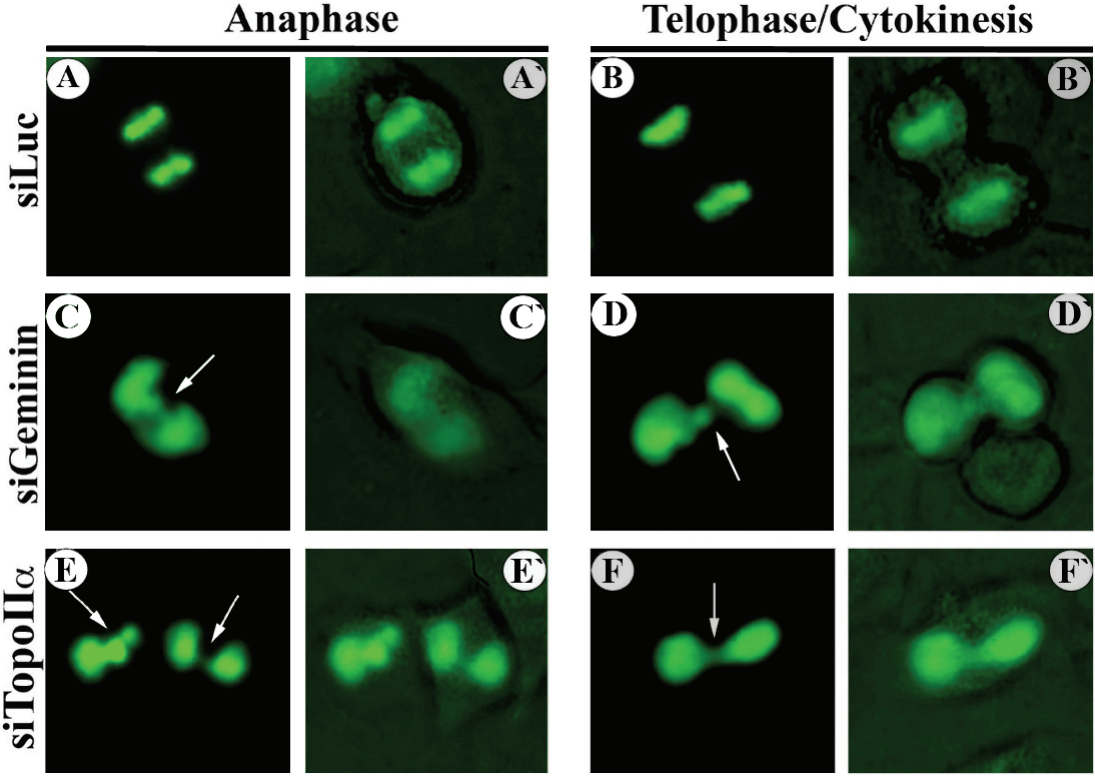
**Geminin silencing induces chromosome bridges**. Human mammary epithelial cell/histone 2B fused to green fluorescence protein (HME/H2B-GFP) protein is an HME cell line that carries an H2B-GFP allele. Examples of live HME/H2B-GFP cells in anaphase **(A and A′, C and C′ and E and E′) **or telophase **(B and B′, D and D′ and F and F′) **72 hours following luciferase control silencing (siLuc) **(A and B′)**, geminin silencing (siGem) **(C and D′) **or topoisomerase IIα silencing (siTopoIIα) **(E and F′)**. Arrows indicate chromosome bridges.

We recently showed that geminin silencing promotes mitotic arrest in HME cells [12]. To elucidate the mechanism whereby this occurs, we generated an HME cell line that carried a histone 2B fused to green fluorescence protein (H2B-GFP) cDNA. Unlike control siLuc-treated cells, geminin silencing induced anaphase (compare Figures 1C and 1C′ to Figures 1A and 1A′) or telophase (compare Figures 1D and 1D′ to Figures 1B and 1B′) chromosome bridges in these cells. Previous studies showed that inhibiting the expression or activity of TopoIIα also promotes the formation of chromosome bridges [48,49]. Indeed, TopoIIα silencing in this HME/H2B-GFP cell line also induced anaphase (Figures 1E and 1E′) or telophase (Figures 1F and 1F′) chromosome bridge formation. Similar data were obtained in HME cells treated in the same manner and stained with DAPI (see Additional files 1A to 1C). This suggests that, like TopoIIα, geminin is required for proper chromosome segregation and that the lack of proper chromosome segregation is what arrests geminin-silenced cells in mitosis [12].

### Geminin interacts with TopoIIα in G_2_/M/early G_1 _phase in HME cells

**Figure 2:**
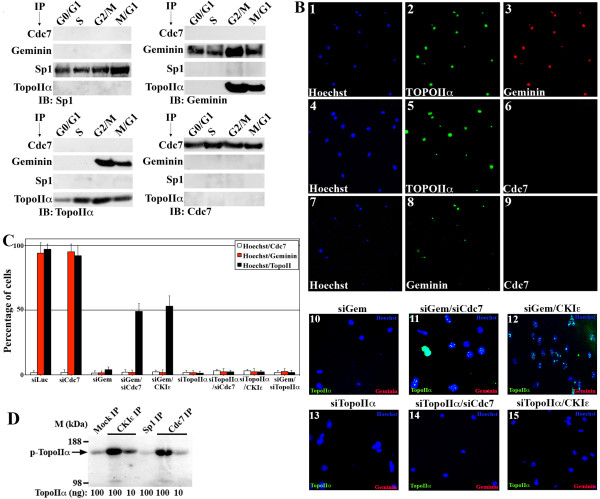
**Geminin interacts with TopoIIα on chromosomes in HME cells**. **(A) **HME cells synchronized in G_0_/G_1_, S, G_2_/M and M/G_1 _were sonicated and immunoprecipitated with the indicated antibodies, followed by immunoblotting with the indicated antibodies. **(B) **HME cells treated with etoposide (10 μM, overnight) were then collected and processed for analysis using trapped in agarose DNA immunostaining (TARDIS) assay. Shown are examples of cells costained with Hoechst 33258 blue, TopoIIα and geminin antibodies (1 to 3), costained with Hoechst 33258 blue, TopoIIα but not Cdc7 antibodies (4 to 6) or costained with Hoechst 33258 blue, geminin but not Cdc7 antibodies (7 to 9). Geminin-silenced HME cells (10), plus cell division cycle 7 silencing (siCdc7) (11) or plus casein kinase Iε (CKIε) overexpression (12) are shown. TopoIIα-silenced HME cells (13) plus Cdc7-silenced (14) or plus CKIε overexpression (15) are also shown. **(C) **Quantification of cells costained with Hoechst 33258 blue and Cdc7 (white bars), geminin (red bars) or TopoIIα (black bars) in TARDIS assays 72 hours after control, Cdc7, geminin, TopoIIα, geminin silencing and Cdc7 silencing (or CKIε overexpression); geminin and TopoIIα silencing; or TopoIIα silencing and Cdc7 silencing (or CKIε overexpression). Values presented are means ± SD. **P ≤ 0.01. **(D) ***In vitro *kinase assay performed on purified TopoIIα using CKIε or Cdc7 immunoprecipitated from HME cells.

To evaluate whether geminin and TopoIIα interact, HME cells synchronized in different parts of the cell cycle (Additional file 2) were sonicated to isolate all cellular proteins, including those on the chromatin. Total cellular proteins were then immunoprecipitated with anti-Cdc7-, anti-geminin-, anti-Sp1 (negative control)- or anti-TopoIIα-specific antibodies. In HME cells, Cdc7, geminin, Sp1 and TopoIIα are all present in all phases of the cell cycle (Figure 2A). The highest level of geminin was observed in G_2_/M cells (Figure 2A), while the lowest level of TopoIIα was observed in G_0_/G_1 _cells (Figure 2A). Cdc7 and Sp1 expression did not change throughout the cell cycle (Figure 2A).

Furthermore, neither Cdc7 nor Sp1 antibodies coimmunoprecipitated geminin or TopoIIα (Figure 2A), and Cdc7 and Sp1 were not coimmunoprecipitated by anti-geminin or anti-TopoIIα antibodies (Figure 2A). Meanwhile, anti-geminin antibody coimmunoprecipitated TopoIIα and anti-TopoIIα antibody coimmunoprecipitated geminin specifically from G_2_/M and M/G_1 _cells (Figure 2A). These data suggest that geminin and TopoIIα form a complex in G_2_/M/early G_1 _cells, to which Cdc7 is not recruited.

### Geminin interacts with TopoIIα on chromosomes in HME cells

To evaluate whether a geminin-TopoIIα interaction occurs on chromosomes, we employed the trapped in agarose DNA immunostaining (TARDIS) assay, which detects TopoIIα on chromosomal arms [47]. HME cells that had been exposed to 10 μM etoposide for 16 hours (to stabilize TopoIIα cleavable complexes) were embedded in agarose-covered microscope slides and lysed to remove cell membrane and soluble proteins. After washing the cells with high salt buffer (1 M NaCl) to remove all noncovalently bound nuclear proteins, the remaining chromosome-protein complexes were studied by using immunofluorescence and Hoechst 33258 blue dye DNA staining.

In control siLuc-treated cells, > 90% of the Hoechst 33258 blue-stained chromosomes were TopoIIα- and geminin-positive (see Figure 2B, 1 to 3, and red and black bars in Figure 2C). Importantly, the same spots on chromosomes that stained for TopoIIα were clearly stained for geminin (Additional file 3A). Although the Cdc7 level rose in geminin-silenced cells (Additional files 3B and 3C), Hoechst 33258 blue-stained TopoIIα- or geminin-positive chromosomes were Cdc7-negative (see Figure 2B, 4 to 6, and Figure 2B, 7 to 9, respectively, as well as white bars in Figure 2C). Surprisingly, geminin silencing abolished TopoIIα chromosome recruitment (Figure 2B, 10, and Figure 2C). Similarly, in TopoIIα-silenced cells, geminin was absent from chromosomes (Figure 2B, 13, and Figure 2C). Since TopoIIα expression was not affected in geminin-silenced cells and *vice versa *(Additional file 3C), these data suggest that geminin and TopoIIα stabilize each other on chromosomes.

Although Cdc7 silencing did not affect TopoIIα or geminin chromosome recruitment (Figure 2C), its cosilencing restored TopoIIα recruitment to chromosomes in geminin-silenced cells (Figure 2B, 11, and Figure 2C), but not geminin recruitment to chromosomes in TopoIIα-silenced cells (Figure 2B, 14, and Figure 2C). These data suggest that Cdc7 upregulation in geminin-silenced cells exerts negative regulation on TopoIIα chromosome localization, perhaps by phosphorylation. In support of this interpretation, the transit overexpression of the positive regulator CKIε that phosphorylates TopoIIα restored the recruitment of TopoIIα to chromosomes in geminin-silenced cells (Figure 2B, 12, and Figure 2C) and not the recruitment of geminin to chromosomes in TopoIIα-silenced cells (Figure 2B, 15, and Figure 2C). Taken together, this information suggests that geminin is required for TopoIIα recruitment to chromosomes and that, while CKIε is an upstream positive regulator of TopoIIα chromosome recruitment, Cdc7 is an upstream negative regulator of TopoIIα chromosome recruitment.

### Cdc7 phosphorylates TopoIIα *in vitro*

To evaluate whether the serine kinase Cdc7 [50] indeed phosphorylates TopoIIα, we used an *in vitro *kinase assay. One milligram of sonicated cycling HME total cell extract was incubated with protein A beads coupled to anti-Cdc7, anti-CKIε or anti-Sp1 (negative control) antibodies. Immunoprecipitated proteins were then incubated for 30 minutes with 100 or 10 ng of purified TopoIIα (TopoGen) in the presence of radioactive ATP. While mock and Sp1 immunoprecipitates did not phosphorylate TopoIIα in this assay (Figure 2D), Cdc7 phosphorylated the 100 ng quantity, and CKIε phosphorylated the 100 and 10 ng quantities, of purified TopoIIα protein (Figure 2D). These data suggest that Cdc7 indeed phosphorylates TopoIIα, at least *in vitro*.

### Cdc7 is upregulated in geminin-silenced cells to enforce the mitotic checkpoint induced in these cells

**Figure 3:**
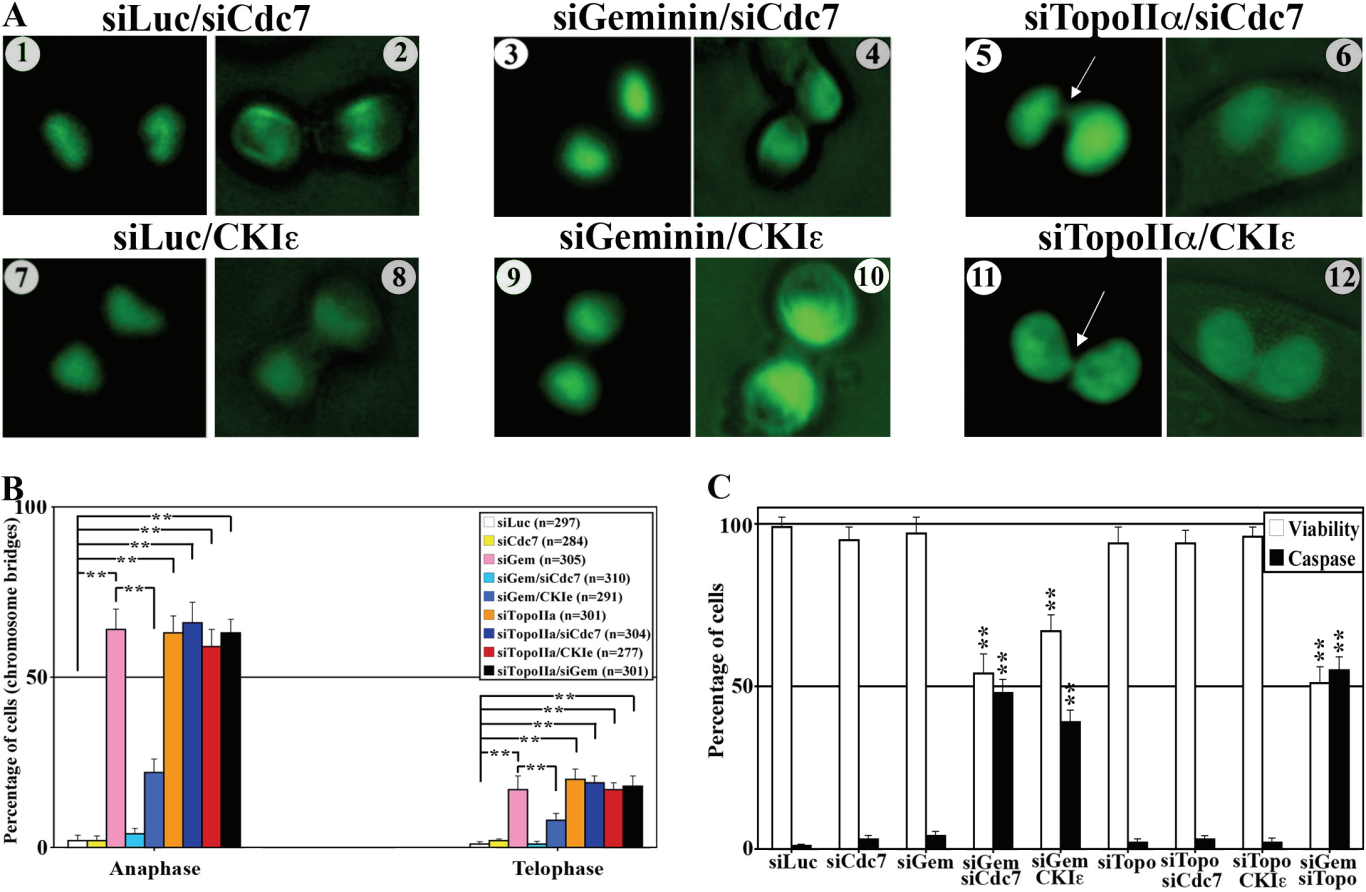
**Cdc7 silencing or CKIε overexpression restores chromosome decatenation in geminin-silenced cells and leads to cell death**. **(A) **Cdc7 silencing in control-silenced (1, 2), geminin (3, 4) or TopoIIα-silenced (5, 6) H2B-GFP HME cells, or CKIε overexpression in control (7, 8), geminin (9, 10) or TopoIIα (11, 12) H2B-GFP HME cells. **(B) **Shown are the percentages of cells with anaphase or telophase chromosomal bridges in cells that were control-silenced (*n *= 297, white bars), Cdc7-silenced (*n *= 284, yellow bars), geminin-silenced (*n *= 305, pink bars), geminin/Cdc7-silenced (*n *= 310, light blue bars), geminin-silenced/CKIε-overexpressing (*n *= 291, medium blue), TopoIIα-silenced (*n *= 301, orange bars), TopoIIα/Cdc7-silenced (*n *= 304, dark blue bars), TopoIIα-silenced/CKIε-overexpressing (*n *= 277, red bars), geminin/TopoIIα-silenced (*n *= 301, black bars). Values presented are means ± SD. **P ≤ 0.01. **(C) **Shown are percentages of viable cells (white bars) or apoptotic cells (with high caspase activity; black bars) following control, Cdc7, geminin, geminin/Cdc7, TopoIIα, TopoIIα/Cdc7 and geminin/TopoIIα silencing, as well as geminin silencing/CKIε overexpression and TopoIIα silencing/CKIε overexpression, as well as geminin/TopoIIα silencing. Values presented are means ± SD. **P ≤ 0.01.

Neither Cdc7 silencing nor CKIε overexpression affected chromosome segregation in control (siLuc) HME/GFP-H2B cells (Figure 3A, 1 and 2, and Figure 3A, 7 and 8, respectively, and Figure 3B) or HME cells (Additional files 1D and 1G, respectively). Geminin but not TopoIIα silencing increased Cdc7 and decreased CKIε expression (Additional files 3B and 3C and data not shown). Cdc7 silencing or CKIε overexpression in geminin-silenced cells restored chromosome segregation stalled in geminin- and not in TopoIIα-silenced HME cells expressing H2B-GFP (Figure 3A, 3 to 6, and Figure 3A, 9 to 12, respectively, and Figure 3B) or in HME cells (Additional files 1E through 1H and Additional files 1F through 1I, respectively). These data reinforce the view that CKIε is a positive regulator and Cdc7 is a negative regulator of TopoIIα chromosome localization and segregation function.

Geminin [12], TopoIIα [34,35] and Cdc7 silencing or CKIε overexpression had minimal effects on S-phase progression or DNA replication based on cell cycle and bromodeoxyuridine incorporation analysis (data not shown). Moreover, while Cdc7 silencing or CKIε overexpression did not block cells from existing mitosis, geminin or TopoIIα silencing did (see low expression of mitotic proteins in geminin-silenced cells in Additional file 3B and [12]), which can explain why these treatments had no effect on HME cell viability or cell death (Figure 3C). However, Cdc7 cosilencing or CKIε overexpression in geminin- and not TopoIIα-silenced cells reduced cell viability and induced cell death (Figure 3C). These data suggest that restoring TopoIIα localization and function by silencing of Cdc7 or overexpression of CKIε in geminin-silenced and/or mitosis-arrested cells induces cell cycle progression followed by cell death. Presumably, cells are unprepared to complete mitosis, perhaps because of the low expression of mitotic proteins observed in these cells (see Additional file 3B and [12]). Finally, we speculate that the cell death observed in geminin- and TopoIIα-silenced cells is due to the activation of p53 in these G_2_/M-arrested cells. These data put Cdc7, like CKIε, upstream of TopoIIα and both Cdc7 and CKIε downstream of geminin with regard to chromosome segregation and mitosis progression.

### Cdc7 upregulation in geminin-silenced cells suppresses TopoIIα chromosome localization and decatenation activity

Next we studied whether geminin is required for TopoIIα decatenation activity. TopoIIα was immunoprecipitated from the chromatin of HME cells silenced from Cdc7, geminin or TopoIIα for 72 hours or exposed to 10 μM doxorubicin or 10 μM etoposide (TopoIIα inhibitors) for 24 hours. To confirm that chromatin-bound TopoIIα was used in these experiments, immunoprecipitated proteins were digested with proteinase K and the DNA was visualized on agarose gel (see Additional file 3D).

**Figure 4:**
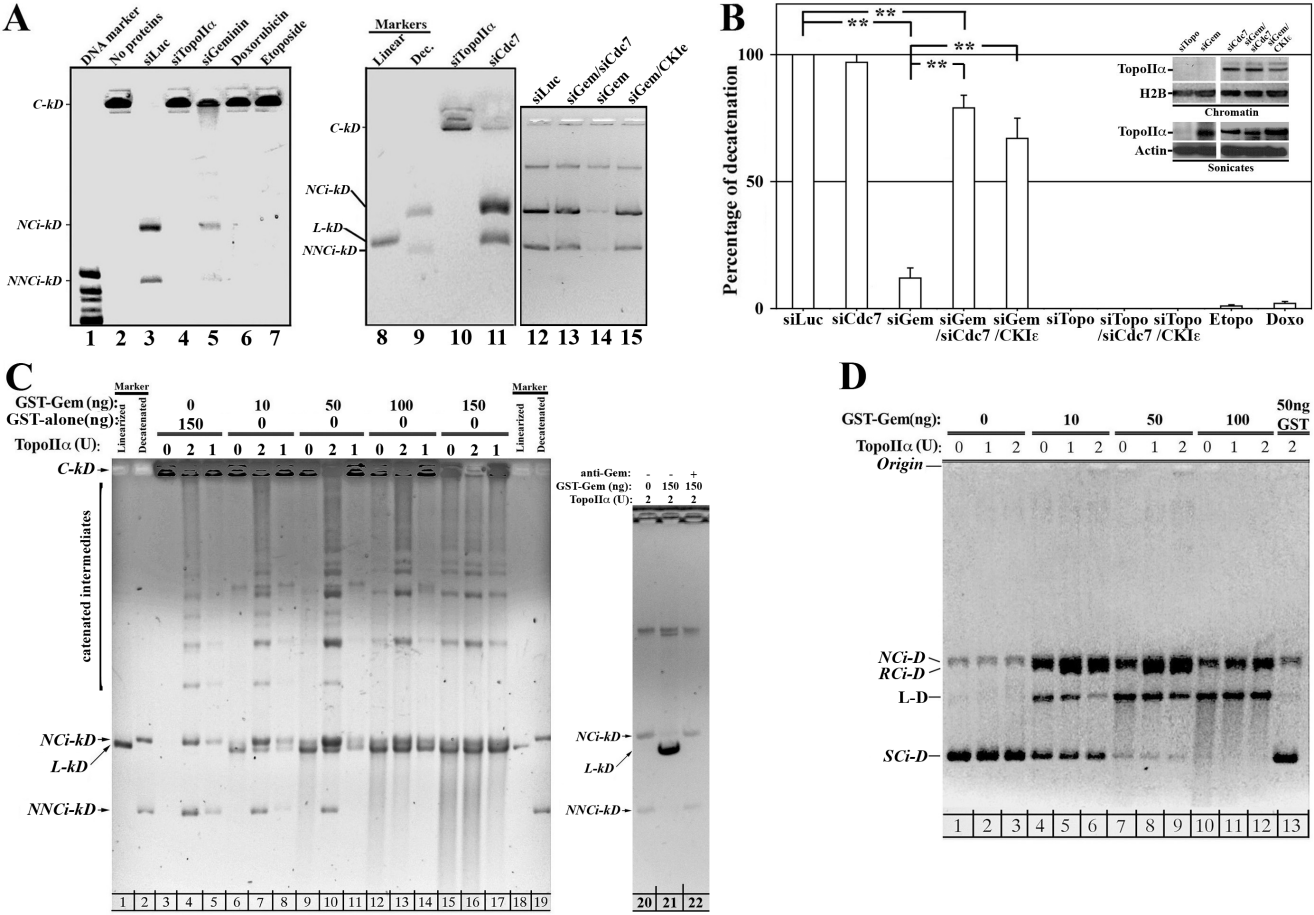
**Geminin at low or high concentrations prevents TopoIIα activity**. **(A) **TopoIIα immunoprecipitated from the chromatin of HME cells 72 hours after transfection with control, geminin, Cdc7 or TopoIIα small interfering RNA (siRNA) or 24 hours after doxorubicin (10 μM) or etoposide (10 μM) treatment were used to decatenate *kinetoplast *(*k*)-*DNA *as a substrate using a TopoGen assay as described in Materials and methods. *NCi-kD *indicates the position of nicked circular decatenated *k-DNA *minicircles and *NNCi-kD *indicates the position of non-nicked circular decatenated *k-DNA *minicircles, while the catenated DNA networks are retained in the wells of the gel (*C-kD*). The markers are 1-kb DNA ladder (lane 1) or linear and decatenated *k-DNA *(lanes 8 and 9). **(B) **Quantification of the reactions shown in **(A)**. Values presented are means ± SD. **P ≤ 0.01. Inset shows the expression of TopoIIα on chromatin (upper) or whole cell (lower) extracts. **(C) **A decatenation assay was carried out using *k-DNA *as the substrate with 0, 1 or 2 U of purified TopoIIα in the presence of 150 ng of glutathione *S*-transferase (GST) alone or 10, 50, 100 or 150 ng of GST-geminin. The reactions on the right are decatenation reactions in the presence of 2 U of TopoIIα alone or with 150 ng of GST-geminin or 150 ng of GST-geminin that was incubated earlier with an excess anti-geminin antibody. **(D) **Relaxation of supercoiled *pBR233 DNA *assay. The plasmid *pBR233 *was incubated with 0, 1 or 2 U of purified TopoIIα in the presence of 50 ng of GST alone or 0, 10, 50 or 100 ng of GST-geminin as described in Materials and methods. *SCi-D*, *RCi-D*, *L-D *and *NCi-D *indicate the positions of supercoiled, relaxed, linear and nicked circular *pBR233 *DNA.

Protein A beads coupled to TopoIIα were then used to decatenate entangled *k-DNA in vitro *using the TopoGen decatenation kit. TopoIIα immunoprecipitated from the control siLuc cells efficiently decatenated *k-DNA *(see nicked circular (*NCi-kD*) and non-nicked circular (*NNCi-kD*) decatenated bands in Figure 4A, lanes 3 and 12). The intensity of these bands was measured using ImageJ software (National Institutes of Health, Bethesda, MD, USA) and taken as 100% (Figure 4B). As expected, no TopoIIα could be immunoprecipitated from the chromatin of TopoIIα-silenced cells (Figure 4B, inset) or TopoIIα-inhibited cells, and thus the immunoprecipitates failed to decatenate *k-DNA *(Figure 4A, lanes 4, 10, 6 and 7, respectively, and Figure 4B). Moreover, TopoIIα was localized to chromatin in Cdc7- and not geminin-silenced cells (Figure 4B, inset). Thus TopoIIα immunoprecipitated from the chromatin of Cdc7-silenced cells efficiently decatenated *k-DNA *(Figure 4A, lane 11, and Figure 4B), whereas TopoIIα immunoprecipitated from the chromatin of geminin-silenced cells had minimal decatenation activity (Figure 4A, lanes 5 and 14, and Figure 4B). Importantly, Cdc7 silencing or CKIε overexpression in geminin-silenced cells restored TopoIIα recruitment to chromatin (Figure 4B, inset) and the immunoprecipitated protein's ability to decatenate *k-DNA *(Figure 4A, lanes 13 and 15, respectively, and Figure 4B). These data reinforce the view that Cdc7 is a negative regulator, and CKIε is a positive regulator, of TopoIIα chromatin localization and decatenation activity.

### High geminin level also inhibits TopoIIα decatenation activity *in vitro*

Next, by using a TopoGen decatenation assay, we sought to determine whether a high geminin level also affects TopoIIα decatenation activity. GST alone (150 ng) was incubated with *k-DNA *in the absence or presence of 1 or 2 U of TopoIIα. No decatenation of *k-DNA *was observed in the reactions containing no TopoIIα (Figure 4C, lane 3). In the presence of 1 or 2 U of purified TopoIIα, *k-DNA *was efficiently decatenated (2 U > 1 U; Figure 4C, lanes 4 and 5). Next, different concentrations of GST-geminin were added to the *k-DNA *in the absence or presence of 1 or 2 U of purified TopoIIα. In the absence of TopoIIα, we noticed that the *k-DNA *was linearized by GST-geminin in a concentration-dependent manner (Figure 4C, lanes 6, 9, 12 and 15). When purified TopoIIα was added to these reactions, decatenation (that is, cleavage and religation) of the *k-DNA *was accomplished in reactions containing 10 and 50 ng of GST-geminin as evidenced by the reappearance of *NCi-kD *and *NNCi-kD *(compare Figure 4C, lanes 7 and 8 to lane 6, and lanes 10 and 11 to lane 9). In the presence of 100 and 150 ng of GST-geminin, however, TopoIIα completely lost its ability to religate *k-DNA *as evidenced by the increased intensity of the linearized bands and the decreased intensity of the decatenated bands (Figure 4C, lanes 12 to 17). These data suggest that, at higher concentrations, geminin prevents the ligation ability of TopoIIα but has no effect on its cleaving activity.

To ascertain that linearization of *k-DNA *by GST-geminin is not entirely due to bacterial nuclease contaminates in this preparation, we incubated *k-DNA *with 2 U of TopoIIα alone or with GST-geminin or GST-geminin previously incubated with anti-geminin antibody. TopoIIα completely decatenated the *k-DNA *(Figure 4C, lane 20). Adding 150 ng of GST-geminin to this reaction again led to *k-DNA *linearization (Figure 4C, lane 21). Importantly, when GST-geminin was first incubated with excess anti-geminin monoclonal antibody and then added to the reaction, almost complete restoration of TopoIIα decatenation activity was observed (Figure 4C, lane 22). Taken together, these findings support the view that geminin possesses nuclease activity, which is in line with our finding that, on a Coomassie blue-stained gel, we could detect a band of only about 25 kDa that corresponded to bacterial purified GST alone and a band of only about 55 kDa that corresponded to GST-geminin (Additional file 3E). However, several alternative explanations are described in the discussion section below.

### High geminin level also inhibits TopoIIα ability to resolve negative supercoiling *in vitro*

As an alternative approach, we asked whether geminin affects TopoIIα's ability to resolve negative supercoiling from the plasmid pBR322. In the absence of TopoIIα, whether 100 ng of GST alone was or was not added to the plasmid pBR322, the DNA appeared to be unaffected as a supercoiled form (*SCi-D*) (Figure 4D, lanes 13 and 1, respectively). Surprisingly, however, adding 1 or 2 U of purified TopoIIα to the last reaction also was unable to relax the plasmid pBP322 (see lack of the relaxed (*RCi-D*) band in Figure 4D, lanes 2 and 3). When a low concentration of GST-geminin (10 ng) was added, the DNA was converted into the nicked form (*NCi-D*) as well as the linear form (*L-D*) (*NCi-D *>*L-D*; Figure 4C, lane 4). Increasing the GST-geminin concentration tilted the reaction toward the linear form (*L-D *>*NCi-D*; see lanes 7 and 10 in Figure 4D).

Interestingly, in the presence of TopoIIα and GST-geminin, the plasmid was relaxed, although the levels of the relaxed form of pBP322 (*RCi-D*) decreased with increasing concentrations of GST-geminin (Figure 4C, compare lanes 11 and 12 to lanes 5 and 6 as well as to lanes 8 and 9). At 2 U, TopoIIα was more efficient in completing religation than at 1 U as measured by the increase in the level of *RCi-D *and the decrease in *L-D *forms (compare lane 6 to lane 5 and lane 9 to lane 8 in Figure 4C). These data suggest that a higher concentration of GST-geminin reduces TopoIIα's ability to complete the relaxation process and prevents the religation of DNA. Several alternative models to explain these data are described in the discussion section below. Taken together, these observations reveal that geminin inhibits TopoIIα decatenation and relaxation activity in a concentration-dependent manner, at least *in vitro*.

### Geminin silencing prevents TopoIIα binding to chromosomes *in vivo*

**Figure 5:**
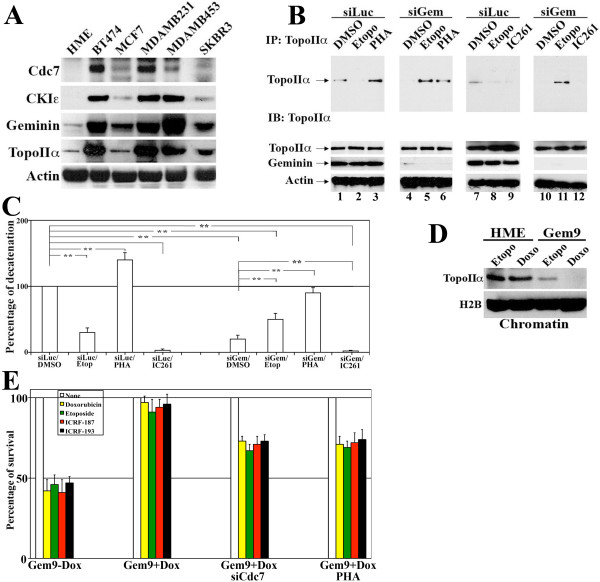
**Geminin overexpression inhibits TopoIIα chromatin localization and activity**. **(A) **Expression of Cdc7, CKIε, geminin and TopoIIα in several breast cancer cell lines. **(B) **Top: TopoIIα immunoprecipitated from the chromatin of MDAMB231 cells following Luc (control) or geminin silencing for 72 hours and treatment with dimethyl sulfoxide (DMSO), etoposide (10 μM), PHA767491 (10 μM) or IC261 (10 μM) during the last 24 hours. Bottom: TopoIIα and geminin from whole cell extracts of the treatments described above. **(C) **Quantification of decatenation using *k-DNA *as the substrate by TopoIIα immunoprecipitated from the chromatin of MDAMB231 treated with the treatments indicated in **(B)**. Values presented are means ± SD. **P ≤ 0.01. **(D) **TopoIIα levels on the chromatin of HME or induced Gem9 (72 hours) following 24-hour exposure to etoposide (etopo, 10 μM) and doxorubicin (doxo, 10 μM). **(E) **The effect of DMSO (none) or 10 μM etoposide, doxorubicin, ICRF-187 or ICRF-193 exposure for 24 hours on the viability of Gem9 cells grown in the presence or absence of doxycycline (2 μg/mL) and Cdc7 siRNA (72 hours) or 10 μM PHA767491 (last 24 hours) as measured using the MTS assay.

Compared to HME cells, geminin, TopoIIα, Cdc7 and CKIε proteins are overexpressed in several estrogen receptor (ER)-positive breast cancer cell lines (for example, MCF7 and BT474) as well as ER-negative breast cancer cell lines (for example, MDAMB231, MDAMB453 and SKBR3) (Figure 5A). The MDAMB231 cell line was chosen to perform the following experiments. Low but detectable levels of TopoIIα were immunoprecipitated from control (siLuc/DMSO)-treated MDAMB231 cell chromatin (Figure 5B, lanes 1 and 7), which efficiently decatenated *k-DNA *in the TopoGen assay (taken as 100%; Figure 5C and Additional file 4 lanes 1 and 7). In contrast, although TopoIIα was detected in whole cell extracts (sonicates) of geminin-silenced MDAMB231 cells (that is, siGem/DMSO lanes in Figure 5B, bottom panels), no detectable protein could be immunoprecipitated from the chromatin of these cells (Figure 5B, lanes 4 and 10), and these immunoprecipitates showed only < 15% decatenation activity in the TopoGen assay (Figure 5C and Additional file 4 lanes 4 and 9).

CKIε (already reduced in geminin-silenced cells; see Additional file 5A) inactivation using the specific inhibitor IC261 (10 μM) significantly decreased the level of TopoIIα immunoprecipitated from the chromatin of control (Figure 5B, lane 9) as well as geminin-silenced (Figure 5B, lane 12) MDAMB231 cells. Both immunoprecipitates failed completely to decatenate *k-DNA *(Figure 5C and Additional file 4 lanes 8 and 10). In contrast, Cdc7 (expression increased in geminin-silenced cells; Additional file 5A) inactivation using the specific inhibitor PHA767491 (10 μM [27,51]) significantly increased the level of TopoIIα immunoprecipitated from the chromatin of control (siLuc)- and geminin-silenced MDAMB231 cells (compare lanes 3 to 1 and 6 to 4, respectively, in Figure 5B). Interestingly, the decatenation activities of both immunoprecipitates were higher than their corresponding DMSO-treated immunoprecipitates (Figure 5C; compare lanes 3 to 1 and 6 to 4 in Additional file 4).

However, because it was proposed earlier that PHA767491 could also target cell division kinase 9 (Cdk9), a kinase involved in the phosphorylation of RNA polymerase II and in the transcriptional regulation of gene expression [27,51-53], we investigated whether gene expression is altered in HME and MDAMB231 cells treated with 10 μM PHA767491 for 24 hours by using a reverse transcriptase polymerase chain reaction assay. In both control and PHA767491-treated HME cells, we detected similar levels of normal expression of cell cycle regulators such as cyclin D1, A and B1; growth factors such as epidermal growth factor and basic fibroblast growth factor; cell surface receptors such as epidermal growth factor receptor and human epidermal growth factor receptor 2; and housekeeping genes such as glyceraldehyde 3-phosphate dehydrogenase, 18S and actin mRNA (Additional file 5B). Similar results were obtained using MDAMB231 cells. Thus, we concluded that, at least in HME or MDAMB231 cells, the effect of PHA767491 on Cdk9 kinase is minimal.

### Geminin overexpression reduces TopoIIα level on chromosomes and induces etoposide resistance

A very low level of TopoIIα was immunoprecipitated from the chromatin of control-treated MDAMB231 cells that were exposed to 10 μM etoposide for 24 hours (Figure 5B, lanes 2 and 8). This immunoprecipitate showed only about 30% decatenation activity (Figure 5C and Additional file 4, lane 2). Interestingly, a significantly higher level of TopoIIα was immunoprecipitated from the chromatin of geminin-silenced MDAMB231 cells (72 hours) that were exposed to 10 μM etoposide during the preceding 24 hours (compare lane 5 to lane 2 and lane 11 to lane 8, respectively, in Figure 5B). These immunoprecipitates showed significantly higher decatenating activity (about 50%) (compare lane 5 to lane 2 in Additional file 4; see also Figure 5C). These data show that endogenous geminin overexpression in breast cancer cell lines such as MDAMB231 prevents the persistence of TopoIIα on chromosomes in a CKIε-dependent (positively) and Cdc7-dependent (negatively) manner, thus reducing the ability of the TopoIIα poison (for example, etoposide) to covalently bind TopoIIα to DNA. This action perhaps decreases these drugs' killing effect.

Indeed, geminin overexpression in Gem9 (HME cell line inducibly (after Dox) overexpressing geminin) led to low TopoIIα levels that could be detected on the chromatin following treatment of these cells with 10 μM etoposide or 10 μM doxorubicin as compared to control HME cells treated the same way (Figure 5D). Furthermore, when uninduced Gem9 cells were exposed to 10 μM TopoIIα drugs (etoposide, doxorubicin, ICRF187 or ICFR193) for 24 hours, their viability deceased by about 50% (Figure 5E). The same treatment had no effect on induced Gem9 cells (Figure 5E), except when Cdc7 expression (using siRNA) or activity (using PHA767491) was decreased, although only partially (Figure 5E). These data suggest that geminin overexpression prematurely releases TopoIIα from chromosomes before these drugs can induce binding to DNA. This leads to the failure of these drugs to poison the enzyme and thus suppresses their cellular toxicity. Suppressing Cdc7 expression or activity actually sensitized geminin-overexpressing cells to TopoIIα drugs, inducing cell death. This is most likely due to the increasing level of TopoIIα on chromatin (see Figure 5B).

### Geminin overexpression triggers TopoIIα premature deSUMOylation and release from chromosomes *in vivo*

**Figure 6:**
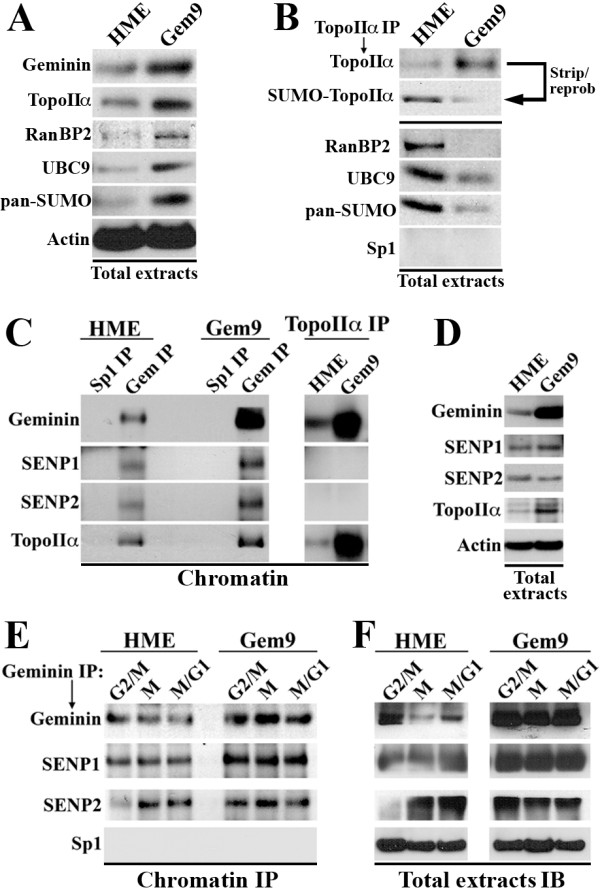
**Geminin overexpression promotes TopoIIα deSUMOylation on chromosomes**. **(A) **Expression in total cell extracts of the indicated proteins in HME or induced Gem9 cells. **(B) **Coimmunoprecipitates of the indicated proteins using anti-TopoIIα antibodies from whole cell extracts. Top image shows reprobing of the TopoIIα immunoprecipitate with anti-Pan SUMO antibody. **(C) **Coimmunoprecipitates of the indicated proteins with anti-Sp1 or anti-geminin antibodies (left) or TopoIIα (right) from the chromatin of HME or induced Gem9 cells. **(D) **Expression of the indicated proteins in HME or induced Gem9 cells (72 hours). **(E) **Coimmunoprecipitates of the indicated proteins with anti-geminin antibody from the chromatin of G_2_/M, M or M/G_1 _HME or induced Gem9 cells (72 hours). **(F) **Expression of the indicated proteins in whole cell extracts of G_2_/M, M or M/G_1 _HME or induced Gem9 cells (72 hours). IP = immunoprecipitation; IB = immunoblotting.

To learn how geminin overexpression prematurely releases TopoIIα from chromosomes, we looked for modifications that target and/or release TopoIIα from chromosomes. It was shown recently that TopoIIα recruitment to chromosomes depends on its state of SUMOylation by the complex RanBP2/Ubc9, while its departure from chromosomes depends on its state of deSUMOylation [39]. We reasoned that geminin overexpression perhaps affects TopoIIα SUMOylation and/or deSUMOylation. Although increased levels of RanBP2, Ubc9, Pan SUMO and TopoIIα were detected in induced Gem9 cells compared to HME cells (Figure 6A), anti-TopoIIα antibody coimmunoprecipitated low levels of RanBP2, Ubc9 and Pan SUMO from induced Gem9 as compared to HME cells (Figure 6B, bottom panels). Consistently, although more TopoIIα was immunoprecipitated from induced Gem9 cells compared to control cells (Figure 6B, top panels), the immunoprecipitated TopoIIα was not SUMOylated in these cells (the blot was reprobed for Pan SUMO; see Figure 6B, top panels).

To our knowledge, no specific deSUMOylating enzyme has yet been identified for TopoIIα. The sentrin-specific proteases SENP1 and SENP2 are two deSUMOylating enzymes with a wide range of substrates [54]. We first evaluated whether geminin interacts with these enzymes. One milligram of HME or induced Gem9 cell chromatin was immunoprecipitated using anti-geminin or anti-Sp1 antibody. SENP1, SENP2 and TopoIIα were coimmunoprecipitated with anti-geminin antibody, but not anti-Sp1 antibody, from the chromatin of HME and induced Gem9 (Figure 6C). Although the expression levels of SENP1 and SENP2 were not changed by geminin overexpression (see Figure 6D), the level of each enzyme immunoprecipitated with geminin antibody from induced Gem9 chromatin was much higher than that immunoprecipitated from HME chromatin (Figure 6C). Furthermore, the geminin-SENP1 complex seems to form on the chromatin of G_2_, M and M/G_1 _cells (Figure 6E) and not on the chromatin of S cells (Additional file 5D, left) in HME and Gem9 cells. In contrast, a geminin-SENP2 complex seems to form on the chromatin of M and M/G_1 _(Figure 6E) but not on the chromatin of G_2_/M (Figure 6E) or S (Additional file 5D, left) of HME cells, whereas in induced Gem9 cells the complex forms on G_2_/M, M and M/G_1 _cells (Figure 6E) but not on S cells (Additional file 5D). These interactions seem to follow the expression of SENP1 and SENP2 (Figure 6F and Additional file 5D, right). While it is possible that the lack of SENP2 in these phases is the reason for the lack of binding between geminin and SENP2, at this moment the lack of binding between geminin and SENP1 in the S phase is less obvious. It is possible that the two proteins are differentially modified in the G_2_/M/early G_1 _phase in such a way that allows them to bind each other that does not exist in the S phase. Another possibility is that the two are separated in space in the S phase but not in the G_2_/M/early G_1 _phase.

Importantly, while TopoIIα expression increased in induced Gem9 cells in comparison to HME cells (Figure 6D), and while anti-TopoIIα antibody immunoprecipitated more TopoIIα from induced Gem9 than HME cell chromatin (Figure 6C, left), anti-TopoIIα did not coimmunoprecipitate SENP1 or SENP2 from HME or induced Gem9 (Figure 6C, left). Taken together, these data show that while geminin binds SENP1 and SENP2 on the chromatin of HME cells and binds more of them on the chromatin in induced Gem9, neither binds to TopoIIα in the presence of either normal (HME) or overexpressed (induced Gem9) levels of geminin. These data show that the low level of TopoIIα detected in etoposide- and doxorubicin-treated cells (see Figure 5D) could be due to geminin overexpression-triggered premature TopoIIα deSUMOylation and departure from chromosomes.

### Geminin overexpression induces survival of DNA damaged cells and leads to aneuploidy in HME cells

Two of the most dire consequences of premature release of TopoIIα from chromosomes by overexpressed geminin, especially before it religates the chromosomes are (1) low efficacy of TopoIIα drugs, for example, doxorubicin or etoposide; and (2) production of damaged chromosomes. Comet assays (natural) that measure DNA tails (the hallmark of *in vivo *double-stranded damaged chromosomes) were used to analyze whether geminin overexpression indeed induces chromosomal breakage by preventing TopoIIα-dependent religation during the decatenation process.

**Figure 7:**
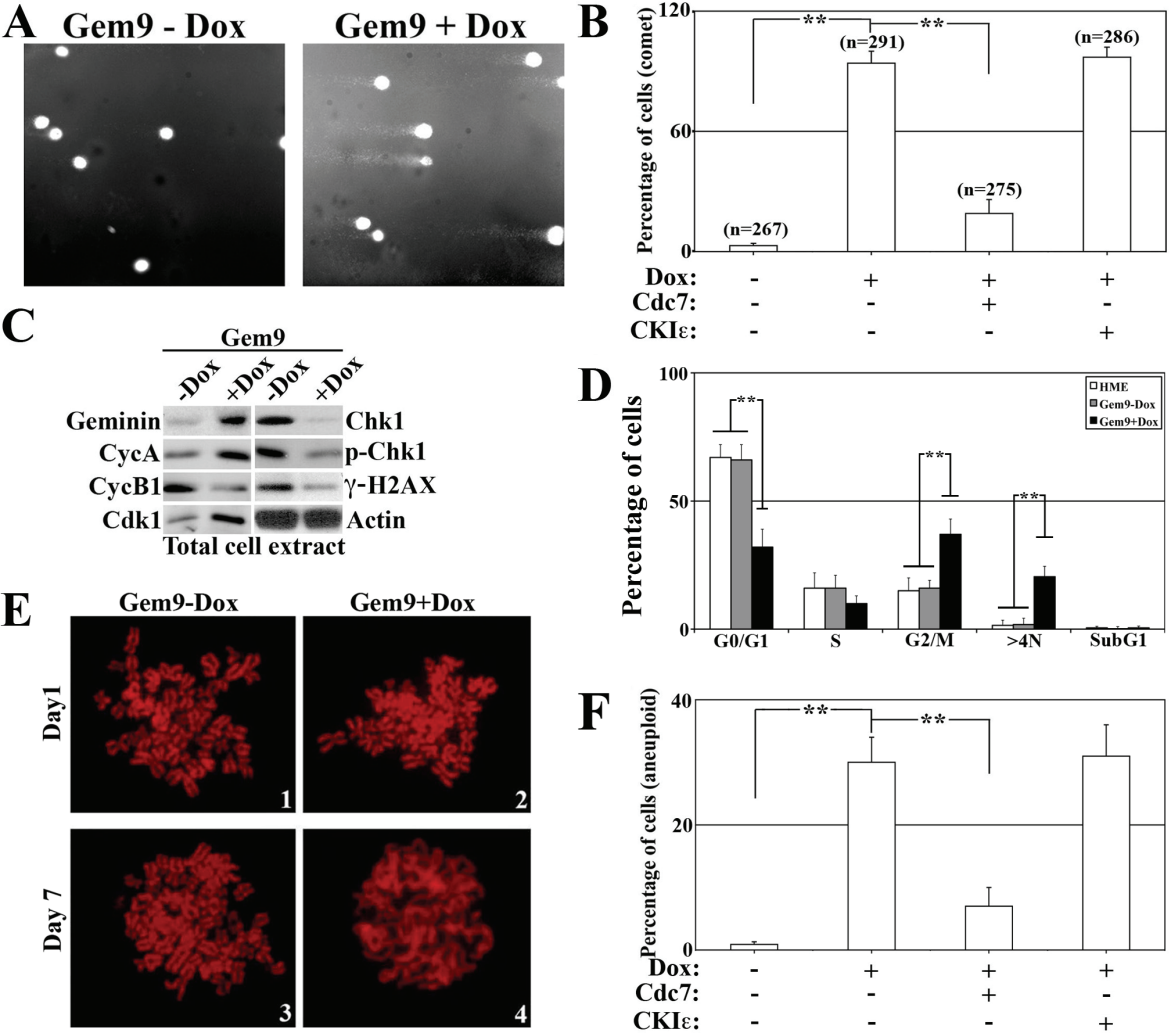
**Geminin overexpression promotes survival of DNA-damaged cells, leading to aneuploidy**. **(A) **Comet assay performed using Gem9 cells grown in the absence (left) or presence (right) of 2 μg/mL doxycycline (Dox) for 72 hours. **(B) **Quantification of the percentages of cells with DNA tails in the comet assay shown in **(A) **in uninduced Gem9, induced Gem9 or induced Gem9 cells transfected with CKIε or Cdc7 cDNA. Values presented are means ± SD. **P ≤ 0.01. **(C) **Expression of cyclin A, cyclin B1, cell division kinase 1 (Cdk1), checkpoint protein 1 (Chk1), phosphorylated Chk1 (p-Chk1) and γ-H2AX in Gem9 cells grown in the presence or absence of 2 μg/mL doxycycline for 72 hours. **(D) **Percentage of G_1_, S, G_2_/M, tetraploid (> 4 N) and apoptotic (sub-G_1_) cells detected using fluorescence-activated cell sorting (FACS) analysis in HME or uninduced or induced Gem9 cells. **(E) **Metaphase spread of uninduced (1 and 3) and induced (2 and 4) Gem9 cells for 1 or 7 days. **(F) **Quantification of percentage of aneuploid cells after eight weeks in uninduced Gem9, induced Gem9 or induced Gem9 transfected with CKIε or Cdc7 cDNA cells detected in metaphase spread analysis. Values presented are means ± SD. **P ≤ 0.01.

While uninduced Gem9 cells showed no DNA tails in this assay (Figure 7A, left, and Figure 7B), induced Gem9 cells (72 hours) showed DNA tails (Figure 7A, right and Figure 7B). Interestingly, overexpression of Cdc7, but not CKIε, in induced Gem9 cells significantly reduced DNA tail formation (Figure 7B). Of note in this comet assay was that induced Gem9 showed DNA tails in > 85% of the cells. Uninduced Gem9 showed DNA tails in about 2% of the cells (Additional file 6A), and geminin, Cdc7 or TopoIIα silencing in uninduced Gem9 cells showed DNA tails in 1% to 2% of the cells (Additional file 6A). These findings seem in line with the notion that the DNA tails are due to damage induced by TopoIIα's premature release from chromosomes, which occurs in geminin-overexpressing, but not geminin-silenced, cells. Accordingly, unlike control-treated cells, geminin- and TopoIIα-silenced, but not Cdc7-silenced, uninduced Gem9 cells were resistant to cell death induced by TopoIIα drugs (Additional file 6B). Taken together, these data show that geminin overexpression triggers DNA damage, most likely by triggering premature release of TopoIIα from chromosomes before it religates DNA.

Moreover, geminin overexpression suppressed the expression and/or activation of the checkpoint protein Chk1 (Figure 7C), as well as the DNA damage-sensing and repair protein γ-H2AX (Figure 7C) [55]. These data indicate that while geminin overexpression promotes DNA damage, the damage is not sensed or repaired and the cell cycle is not arrested as would be the case in cells with a normal level of geminin. Instead, geminin overexpression accelerated the cycle as measured using FACS analysis (Figure 7D). Since geminin-overexpressing cells also show increased levels of mitosis-inducing proteins, for example, cyclin A and Cdk1 (Figure 7C), it would be expected that geminin-overexpressing cells, although damaged, would continue to cycle. This could explain, at least in part, the inability of TopoIIα drugs to induce cell death in induced Gem9 (see Figure 5E) and may imply that geminin overexpression triggers chromosomal abnormalities such as aneuploidy.

Indeed, in our FACS analysis, we detected a high percentage of induced Gem9 cells with > 4 N DNA content as compared to uninduced Gem9 (Figure 7D). Moreover, in analysis of metaphase chromosome spread stained with Giemsa of uninduced, induced or induced but transfected with Cdc7 or CKIε Gem9 cells, we found that while < 1% of uninduced Gem9 cells were aneuploid, while about 30% of induced Gem9 (for eight weeks; that is, about 50 cell divisions) showed aneuploidy. Interestingly, overexpression of Cdc7, but not CKIε, significantly reduced the number of aneuploid cells (Figure 7F).

### Geminin overexpression inhibits TopoIIα activity *in vivo*

To evaluate whether geminin overexpression indeed inactivates TopoIIα *in vivo*, we studied chromosome condensation using metaphase spread. Uninduced or induced Gem9 (for 1, 7 or 28 days) were treated for one hour with the spindle microtubule depolymerizing drug colcemid, followed by metaphase spread and PI staining. While chromosome condensation was visualized under a fluorescence microscope in uninduced and induced Gem9 cells at 1 day (Figure 7E, lanes 1 and 2), at 7 days (Figure 7E, lanes 3 and 4) and at 28 days (not shown), induced Gem9 chromosomes were uncondensed (alternatively, decondensed; Figure 7E, 4), whereas uninduced Gem9 chromosomes were still condensed (Figure 7E, 3). These data suggest that geminin overexpression also inactivates TopoIIα *in vivo*.

On the basis of all of these data, we propose that geminin affects TopoIIα chromosome localization (see TARDIS assay results in Additional file 6D) and activity in a CKIε- and/or Cdc7-dependent manner and that its overexpression induces the formation of aneuploid cells (and does not induce chromosome bridges; see Additional file 6C) by prematurely releasing TopoIIα from chromosomes after it cleaves DNA and before it religates it. These effects could contribute to the generation of aggressive breast cancer cells that are resistant to TopoIIα poison drugs.

## Discussion

Chromosome decatenation and/or segregation and cell division are coordinated in the cell cycle of all organisms, from bacteria to humans. In human cells, TopoIIα is involved in chromosome decatenation, condensation and segregation [48]. Geminin's binding to TopoIIα on mitotic chromosomes and enhancing of its decatenation activity clearly show that geminin's physical and functional interaction with TopoIIα is essential to coordinate chromosome decatenation and/or segregation with cell division. Considering geminin's role in DNA replication, it is possible to suggest that geminin stimulates TopoIIα interaction and helps disentangle the freshly replicated DNA. The negative supercoiling (unwinding) generated at the initiation of replication at ORIs [56,57] and the positive supercoiling (overwinding) generated ahead of the replication fork during replication elongation [58-61] must be resolved to facilitate strand separation. It is possible that through the interaction of geminin and TopoIIα, geminin loads onto or stabilizes TopoIIα on chromosomes and thus increases the level of DNA-bound TopoIIα and the effective rate of decatenation and relaxation of the newly made sister duplexes [62,63].

Since chromosome condensation, alignment at the metaphase plate and movement toward the poles [64] occurred relatively normally in geminin-silenced cells (Figure 1 and Additional file 1), the spindle and associated molecular motors must function correctly in the absence of geminin. Likewise, the normal attachment of the chromosomes to the metaphase plate and lack of checkpoint activation that monitors spindle tension [65] in geminin-silenced cells suggest that kinetochores are also unaffected by geminin silencing. It is thus possible to propose that the primary function for the geminin-TopoIIα complex is to resolve chromosome complexities and that, in the absence of geminin, an increase in the number and complexity of knotted replication bubbles would increase the number of nodes in the catenane [56] that arrest segregation of the freshly replicated DNA molecules [56], leading to chromosome bridges and mitotic arrest [12,66]. Our present study supports this proposition. Several proteins with wide varieties of functions (such as the bacterial condensin-like protein MukB [67], the *Drosophila *condensin protein Barren [68] and the bacterial SeqA protein that prevents overinitiation of chromosome replication [69]) have been shown to function in a similar manner in which they interact and/or stimulate the relaxation and decatenation activities of TopoIIα (TopoIV in bacteria). Similar to geminin silencing, mutations in these genes prevent effective separation of sister chromatids during anaphase because of the suppression of TopoIIα function. In future studies, it will be important to search for other components in this geminin-TopoIIα complex that regulate chromosome decatenation. This work will be necessary to better understand the molecular mechanism of proper decatenation/segregation during mitosis.

One of the interesting and unexpected aspects of the present study is the fact that GST-geminin could induce linearization of *k-DNA *as well as *pBP322 *plasmid. As mentioned above, we cannot rule out bacterial nuclease contaminant in the GST-geminin preparation as the source of the apparent DNA linearization in both assays. However, while this might be possible in the *pBP322 *reaction, it is hard to imagine that this is the case in the *k-DNA *reaction. The *k-DNA *used consisted of interlocked minicircles (mostly 2.5 kb) that form extremely large networks of high molecular weight. Unless cut and religated specifically by TopoIIα during the decatenation process, these networks fail to enter the gel. Assuming that a protein other than geminin cleaved the DNA, it must have been pulled down specifically by GST-geminin and not by GST alone. This would make it a partner and not contaminant. However, bacteria do not express geminin. Therefore, a human homolog of this nuclease must exist and would be worth cloning in the future. Alternatively, it is possible that geminin at higher concentrations binds and masks a TopoIIα ligation-inducing domain, if it exits. This could explain the fact that only at much higher concentrations in these assays did geminin prevent religation, but not cleaving activity, of TopoIIα. Finally, it is possible that geminin itself has nuclease activity. Geminin is a coiled-coil protein [70], and many coiled-coil proteins, such as the Werner syndrome protein WRN [71], are known to have nuclease activity. In support of the latter assumption, the fact that incubating GST-geminin with anti-geminin antibody before the reaction restored TopoIIα's ability to decatenate the *k-DNA *(Figure 4C). At the moment, we are unable to distinguish between these possibilities but have future plans to investigate which is valid.

Phosphorylation of TopoIIα on S1106 is important in TopoIIα translocation to chromosomes, DNA decatenation, formation of drug-stabilized DNA cleavable complex and modulation of drug sensitivity [40,41]. CKIε is the only known kinase that targets this site *in vitro *and *in vivo *[40,41]. The facts that CKIε overexpression restored chromosome decatenation and/or segregation that had stalled in the geminin-silenced cells and that geminin overexpression upregulated CKIε expression suggest a positive molecular link by which geminin controls TopoIIα chromosome localization and function. The facts that geminin overexpression decreased Cdc7 expression, that Cdc7 silencing restored stalled chromosome decatenation and/or segregation (that is, chromosome bridges) [72] in the geminin-silenced cells, that Cdc7 overexpression reduced chromosome breakage and aneuploidy induced by geminin overexpression and that Cdc7 phosphorylated TopoIIα, at least *in vitro*, suggest that Cdc7 is a negative molecular link between geminin and TopoIIα chromosome localization and function. It will be important in future studies to investigate whether Cdc7 also phosphorylates TopoIIα *in vivo *and on which sites, what are the upstream kinases and/or conditions that activate Cdc7 to phosphorylate TopoIIα and what is their relation to geminin.

TopoIIα SUMOylation is inhibited and/or decreased in geminin-overexpressing cells. It is possible that geminin overexpression prevents TopoIIα SUMOylation by decreasing its binding to the SUMOylating complex RanBP2/Ubc9. Alternatively, it is possible that in normal cells, one function of geminin is to bind and/or recruit the deSUMOylating enzymes SENP1 and SENP2 to chromosomally bound TopoIIα and to facilitate its deSUMOylation and release from chromosomes after chromosome decatenation is completed. In geminin-overexpressing cells, this could be accelerated by the fact that geminin recruits more of the enzymes and/or recruits them earlier to TopoIIα, thus leading to premature deSUMOylation and release of TopoIIα from chromosomes before the ligation step. It is also possible that this is simply the result of a dominant negative effect exerted by overexpressed geminin. Whatever the reason is, this could contribute to the generation of DNA damage and low efficiency of TopoIIα-directed drugs. At present, we are investigating whether SENP1 and/or SENP2 are indeed TopoIIα deSUMOylating enzymes; whether a molecular link between geminin-induced TopoIIα phosphorylation, SUMOylation and deSUMOylation exists; and whether using inhibitors of deSUMOylating enzymes in combination with TopoIIα-directed drugs could be used to treat breast cancers with high geminin levels.

It is intriguing that geminin overexpression suppressed cell death induced by two different types of TopoIIα drugs. It has been proposed that cells with low levels of TopoIIα respond better to the types of drugs that interfere with the catalytic activity of the enzyme (for example, ICRF187 and ICRF193), while cells with high TopoIIα levels are most resistant [73,74]. This could explain why, compared to uninduced Gem9 cells, induced Gem9 cells were resistant to these drugs, since geminin overexpression reduced the level of TopoIIα on the chromatin. The other types of TopoIIα drugs (for example, etoposide and doxorubicin) have the potential to induce DNA DSBs by stabilizing TopoIIα on DNA and prevent its religation activity during chromosome decatenation [73,74]. It has also been proposed that these drugs induce a DSB for every drug-stabilized TopoIIα enzyme. Thus sensitivity to this type of drugs increases with the level of the chromosome-bound TopoIIα. Low chromosome-bound TopoIIα was detected in cells expressing endogenously (for example, MDAMB231) or exogenously (induced Gem9), so overexpression of geminin could explain resistance to this type of drug as well. This important aspect of our study implies that the efficacy of all types of TopoIIα-directed drugs should increase if combined with geminin inhibitors.

Our previously published results [12] and those presented in this study are in principle agreement with those published recently by Zhu *et al*. [75]. Those authors claimed that selective killing of cancer cells could be achieved by inhibiting geminin activity. Whereas they claimed that normal cells depleted of geminin continue to proliferate normally [75], we showed earlier that geminin silencing inhibited progression of immortalized HME cells from the M to G_1 _phase with minimal effect on S-phase progression. Furthermore, they proposed that cancer cells depleted of geminin specifically rereplicate their genomes and that their nuclei became giant and underwent apoptosis [75]. However, we proposed that geminin has a fundamental cytokinetic function, whereas its S phase is redundant. This discrepancy could be due to differences in the cell types and/or the techniques used. Another possible reason for this incongruity is that the system cells used in the Zhu *et al*. study continued to express cyclin A [75], while in the HME cells we observed no cyclin A expression in geminin-silenced cells [12]. It would be interesting in future studies to determine whether, in breast cancer cell lines, for example, geminin silencing also induces rereplication as reported by Zhu *et al*. [75]. However, we doubt this will be the finding, because in a future publication (unpublished data, W. M. ElShamy) we will show that continuous geminin silencing (with three different small hairpin RNA) inhibits the proliferation of MDAMB231 cells *in vitro *as well as tumor formation in a mouse xenograft model. Also in contrast to the data presented by Zhu *et al*. [75], in our work it is geminin overexpression, not its silencing in HME cells, that triggers the formation of cells containing > 4 N DNA content *in vitro*. Furthermore, our work shows tetraploid and/or aneuploid karyotyping and giant nuclei both *in vitro *and in a mouse xenograft model in geminin overexpressing cells. Given these apparent differences, only our overall conclusions are in accord with those of Zhu *et al*. [75]. We also propose that inhibiting geminin expression and/or activity should selectively kill cancer cells overexpressing geminin (unpublished data, W. M. ElShamy).

Decreased repair of chromosomal DSBs can lead to genome instability, including mutation, translocation and aneuploidy, all of which are hallmarks of many cancers [76-79]. Interestingly, specific Chk1 and H2AX phosphatase upregulation in geminin overexpression led to their inactivation in Gem9 cells (see Results section).

Taken together, our present findings suggest that geminin overexpression induces the formation of aneuploid, aggressive and drug-resistant breast cancer cells (see model in Additional file 7). Geminin silencing prevents decatenation because it blocks TopoIIα's access to chromosomes and its function therein (see model in Additional file 7). Thus, in combination, our data provide an intriguing molecular explanation for the high percentage of patients in whom TopoIIα-directed treatment fails. We propose that etoposide, doxorubicin or any other drugs that target TopoIIα function will be more beneficial when combined with anti-geminin chemotherapeutic agents.

## Conclusions

In summary, our data provide strong evidence that geminin plays a critical role in mitotic chromosome decatenation and/or segregation. The role of geminin in these processes reflects its ability to influence TopoIIα chromosome localization and activity during the G_2_/M/early G_1 _phase. The specific timing of geminin's association with chromosomes and its regulation of TopoIIα chromosome localization and function in the G_2_/M/early G_1 _phase fit the role of geminin in the induction of proper cytokinesis that we proposed earlier [12]. Our results have established a significant role for geminin via its functional and physical interactions with Cdc7 and/or CKIε and TopoIIα, respectively, in the complex process of mitotic chromosome segregation and execution of proper cytokinesis. As such, geminin represents an extremely attractive target for chemotherapy interventions for aggressive breast cancer, either alone or in combination with TopoIIα drugs.

Our findings have wide significance in providing new insight into how geminin could be involved in gene amplification and translocation in cancer. We suggest that lack of religation during the decatenation cycle by prematurely releasing TopoIIα from the decatenation sites can lead to illegitimate repair and genome rearrangement. Our data also lead us to question previous assumptions concerning the existence of a checkpoint for preventing cells with an incompletely replicated and/or segregated genome from entering mitosis and becoming aneuploid.

## Abbreviations

Cdc7: cell division cycle 7; Cdk1: cell division kinase 1; Chk1: checkpoint protein 1; CKIε: casein kinase Iε; H2B-GFP: histone 2B fused to green fluorescence protein; kDa: kilodalton; TARDIS: trapped in agarose DNA immunostaining; SENP: sentrin-modified protein; TopoIIα: topoisomerase IIα.

## Competing interests

The authors declare that they have no competing interests; however, WME has submitted a patent application for the use of anti-geminin along with TopoIIalpha drugs to treat geminin overexpressing breast tumors.

## Authors' contributions

LG, RM, YS and NM performed the experiments. WME designed, performed and interpreted the experiments and wrote the manuscript. LG, RM, YS, NM and WME read and approved the final manuscript.

## Supplementary Material

Additional file 1 **Geminin silencing induces chromosome bridges**. Unlike control silenced (siLuc) human mammary epithelial (HME) cells **(A)**, geminin silencing (siGem) **(B) **and topoisomerase IIα silencing (siTopoIIα) **(C) **induced chromosome bridge formation. Cell division cycle 7 silencing (siCdc7) **(D) **and casein kinase Iε silencing (siCKIε) overexpression **(G) **did not affect chromosome segregation in HME cells. However, siCdc7 or CKIε overexpression restored chromosome segregation in siGem cells **(E) **and **(H)**, respectively, and not siTopoIIα cells **(F) **and **(I)**, respectively. Click here for file

Additional file 2 **Synchronization of HME cells**. HME cells were synchronized using growth factor starvation (72 hours) and then were taken as G_0_/G_1 _phase **(A**) following the 72 hr. After addition of growth factors, cells reached S phase 16 hours later **(B)**, G_2_/M phase 22 hours later **(C) **and M/G_1 _phase 28 hours later **(D)**. Click here for file

Additional file 3 **Expression of several proteins in siGem cells**. **(A) **Trapped in agarose DNA immunostaining assay-processed HME cells stained with Hoechst 33258 blue, TopoIIα and geminin. **(B) **Expression of selected proteins in HME cells 72 hours following transfection with control siLuc or geminin small interfering RNA. pChk1, phosphorylated checkpoint protein 1. **(C) **Expression of Cdc7, geminin or TopoIIα in HME cells depleted by control siLuc, siGem, siCdc7 or siTopoIIα. **(D) **Analysis of DNA immunoprecipitated by anti-TopoIIα antibody from MDAMB231 cells after luciferase (control) or geminin silencing for 72 hours and the treatments indicated during the last 24 hours. DMSO, dimethyl sulfoxide; IP, immunoprecipitation; PHA, PHA767491. **(E) **Analysis of glutathione *S*-transferase (GST) alone or GST-geminin purification using Coomassie blue stain. Click here for file

Additional file 4 **Geminin silencing abrogates TopoIIα activity**. Decatenation of *k-DNA *using TopoIIα immunoprecipitated from control or geminin-silenced cells for 72 hours. In both cases, the cells were treated with solvent, 10 μM TopoIIα inhibitor etoposide, 10 μM CKIε inhibitor IC261 or 10 μM Cdc7 inhibitor PHA767491. Click here for file

Additional file 5 **Expression of several proteins in geminin-silenced or geminin-overexpressing cells**. **(A) **Expression of the indicated proteins in MDAMB231 cells following geminin silencing detected using immunoblotting. **(B) **Expression of the indicated mRNA in control or PHA767491-treated cells detected using RT-PCR. EGF, epidermal growth factor; EGFR, epidermal growth factor receptor; GAPDH, glyceraldehyde 3-phosphate dehydrogenase; bFGF, basic fibroblast growth factor; ErB2, human epidermal growth factor receptor 2. **(C) **Expression of the indicated proteins in uninduced or induced (72 hours) Gem9 cells after detection using immunoblotting. Dox, doxycycline. **(D) **Coimmunoprecipitates of the indicated proteins with anti-geminin antibody from the chromatin of S-phase HME or induced Gem9 cells (72 hours) (left) or expression of the indicated proteins in whole cell extracts of S-phase HME or induced Gem9 cells (72 hours). IP, immunoprecipitation; IB, immunoblotting; SENP1 and SENP2, sentrin-specific protease enzymes 1 and 2. Click here for file

Additional file 6 **Effects of geminin silencing or overexpression on DNA damage and drug response**. **(A) **Comet assay comparing induced Gem9 cells to uninduced Gem9 cells following luciferase, geminin, Cdc7 or TopoIIα silencing. **(B) **The effect of the TopoIIα drugs doxorubicin, etoposide, ICRF187 and ICRF193 on the viability of induced Gem9 or uninduced Gem9 when luciferase, geminin, TopoIIα and Cdc7 were silenced. **(C) **Numbers of anaphase or telophase bridges in geminin-silenced, induced Gem9, induced Gem9 overexpressing Cdc7 or CKIε. **(D) **Percentage of cells stained for Hoechst 33258 blue and Cdc7, geminin or TopoIIα in luciferase- or geminin-silenced cells, uninduced or induced Gem9 cells, or induced Gem9 when Cdc7 or CKIε was overexpressed. Click here for file

Additional file 7 **Geminin effects on TopoIIα chromosome localization and function**. Schematic model of the proposed effects of geminin in normal cells (left), geminin-silenced cells (middle) and geminin-overexpressing cells (right). Click here for file
