# Supplementary figures and images for: Geminin overexpression prevents the completion of topoisomerase IIα chromosome decatenation, leading to aneuploidy in human mammary epithelial cells
Source: Breast Cancer Res. 2011 May 19;13(3):R53. doi: 10.1186/bcr2884 (PMC3218940; doi:10.1186/bcr2884)

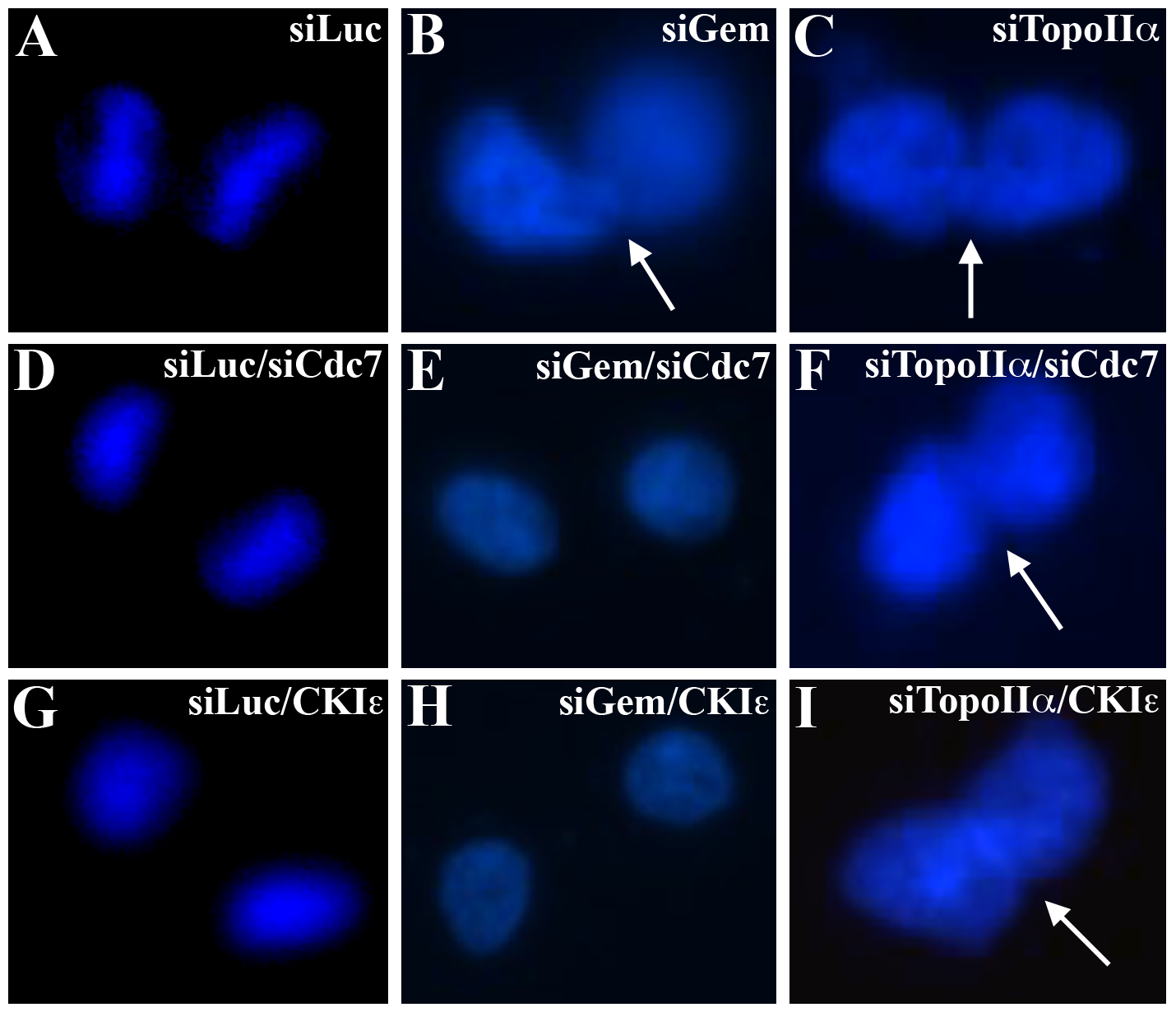

Supplement: Additional file 1 — Geminin silencing induces chromosome bridges. Unlike control silenced (siLuc) human mammary epithelial (HME) cells (A), geminin silencing (siGem) (B) and topoisomerase IIα silencing (siTopoIIα) (C) induced chromosome bridge formation. Cell division cycle 7 silencing (siCdc7) (D) and casein kinase Iε silencing (siCKIε) overexpression (G) did not affect chromosome segregation in HME cells. However, siCdc7 or CKIε overexpression restored chromosome segregation in siGem cells (E) and (H), respectively, and not siTopoIIα cells (F) and (I), respectively. [file bcr2884-S1.TIFF]

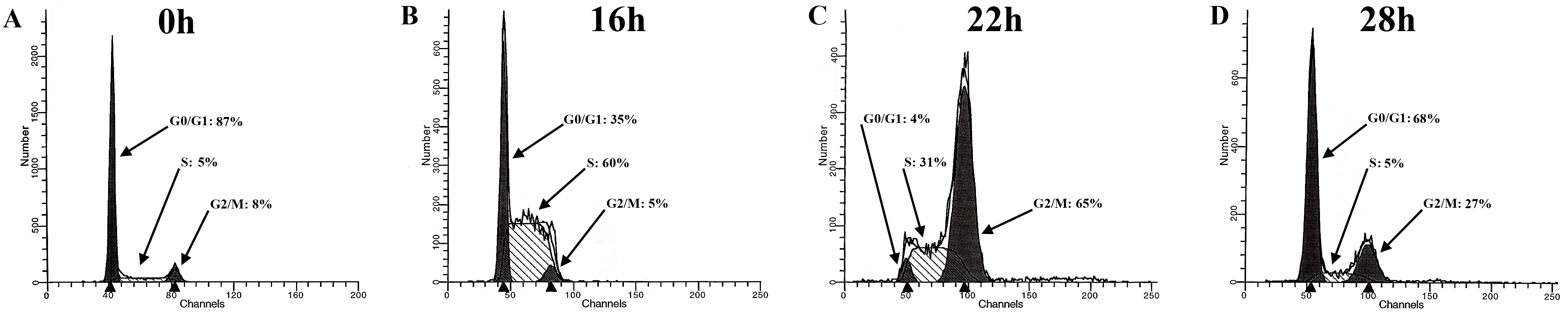

Supplement: Additional file 2 — Synchronization of HME cells. HME cells were synchronized using growth factor starvation (72 hours) and then were taken as G0/G1 phase (A) following the 72 hr. After addition of growth factors, cells reached S phase 16 hours later (B), G2/M phase 22 hours later (C) and M/G1 phase 28 hours later (D). [file bcr2884-S2.TIFF]

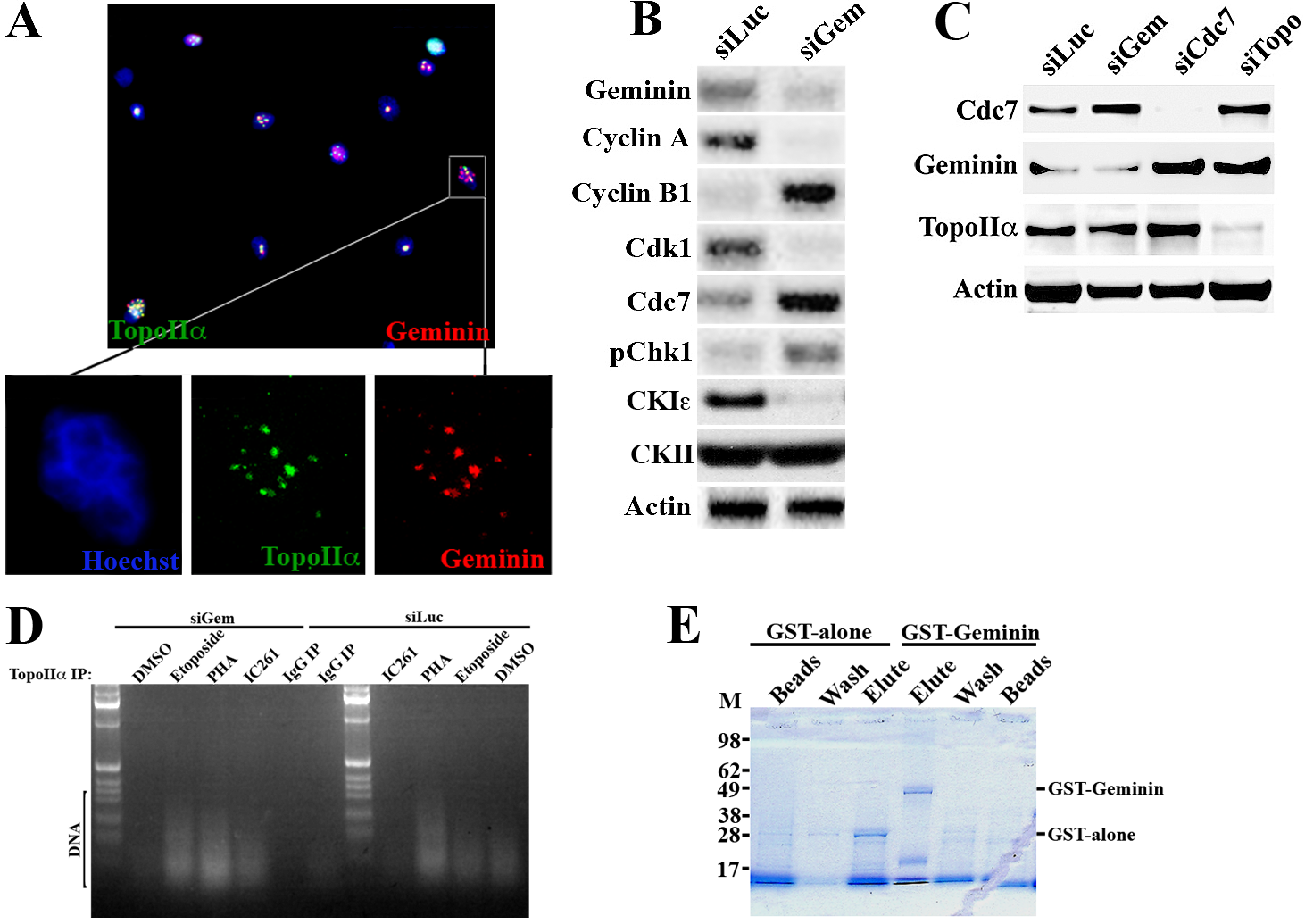

Supplement: Additional file 3 — Expression of several proteins in siGem cells. (A) Trapped in agarose DNA immunostaining assay-processed HME cells stained with Hoechst 33258 blue, TopoIIα and geminin. (B) Expression of selected proteins in HME cells 72 hours following transfection with control siLuc or geminin small interfering RNA. pChk1, phosphorylated checkpoint protein 1. (C) Expression of Cdc7, geminin or TopoIIα in HME cells depleted by control siLuc, siGem, siCdc7 or siTopoIIα. (D) Analysis of DNA immunoprecipitated by anti-TopoIIα antibody from MDAMB231 cells after luciferase (control) or geminin silencing for 72 hours and the treatments indicated during the last 24 hours. DMSO, dimethyl sulfoxide; IP, immunoprecipitation; PHA, PHA767491. (E) Analysis of glutathione S-transferase (GST) alone or GST-geminin purification using Coomassie blue stain. [file bcr2884-S3.TIFF]

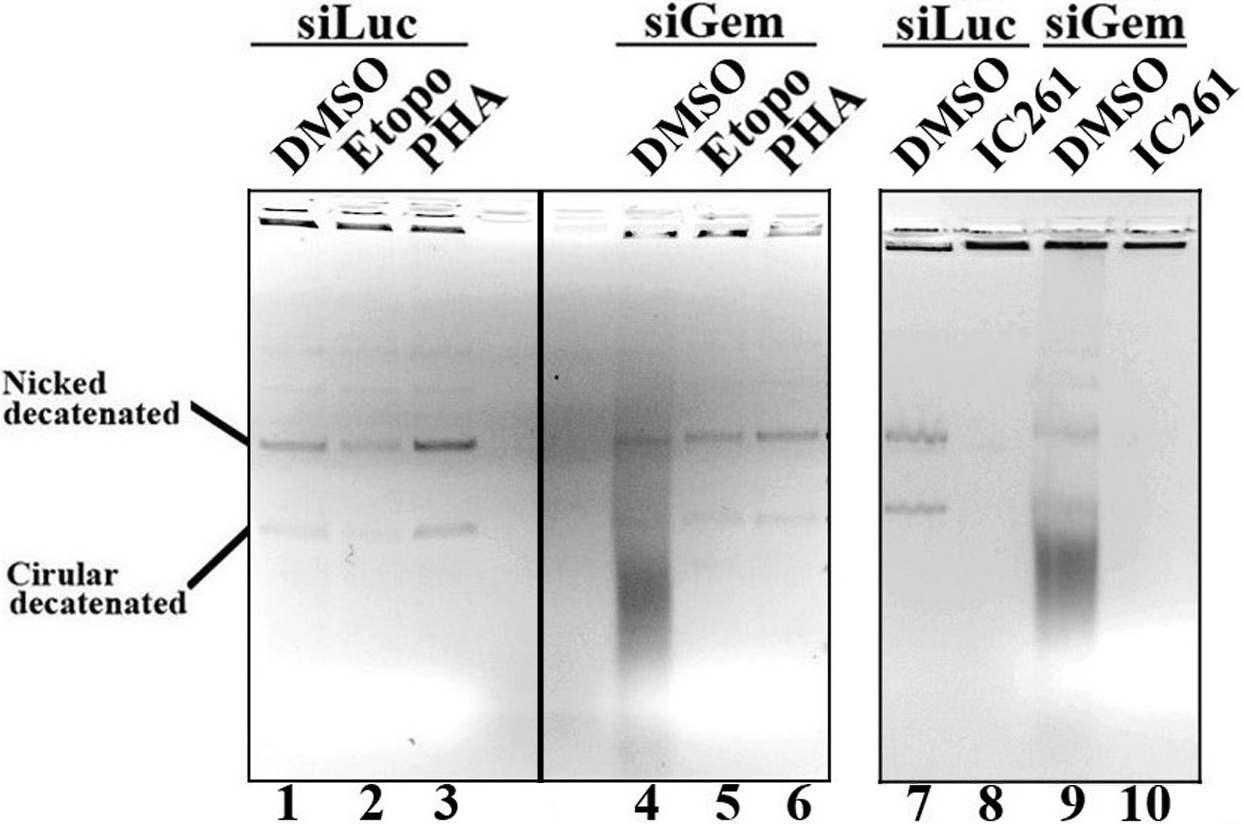

Supplement: Additional file 4 — Geminin silencing abrogates TopoIIα activity. Decatenation of k-DNA using TopoIIα immunoprecipitated from control or geminin-silenced cells for 72 hours. In both cases, the cells were treated with solvent, 10 μM TopoIIα inhibitor etoposide, 10 μM CKIε inhibitor IC261 or 10 μM Cdc7 inhibitor PHA767491. [file bcr2884-S4.TIFF]

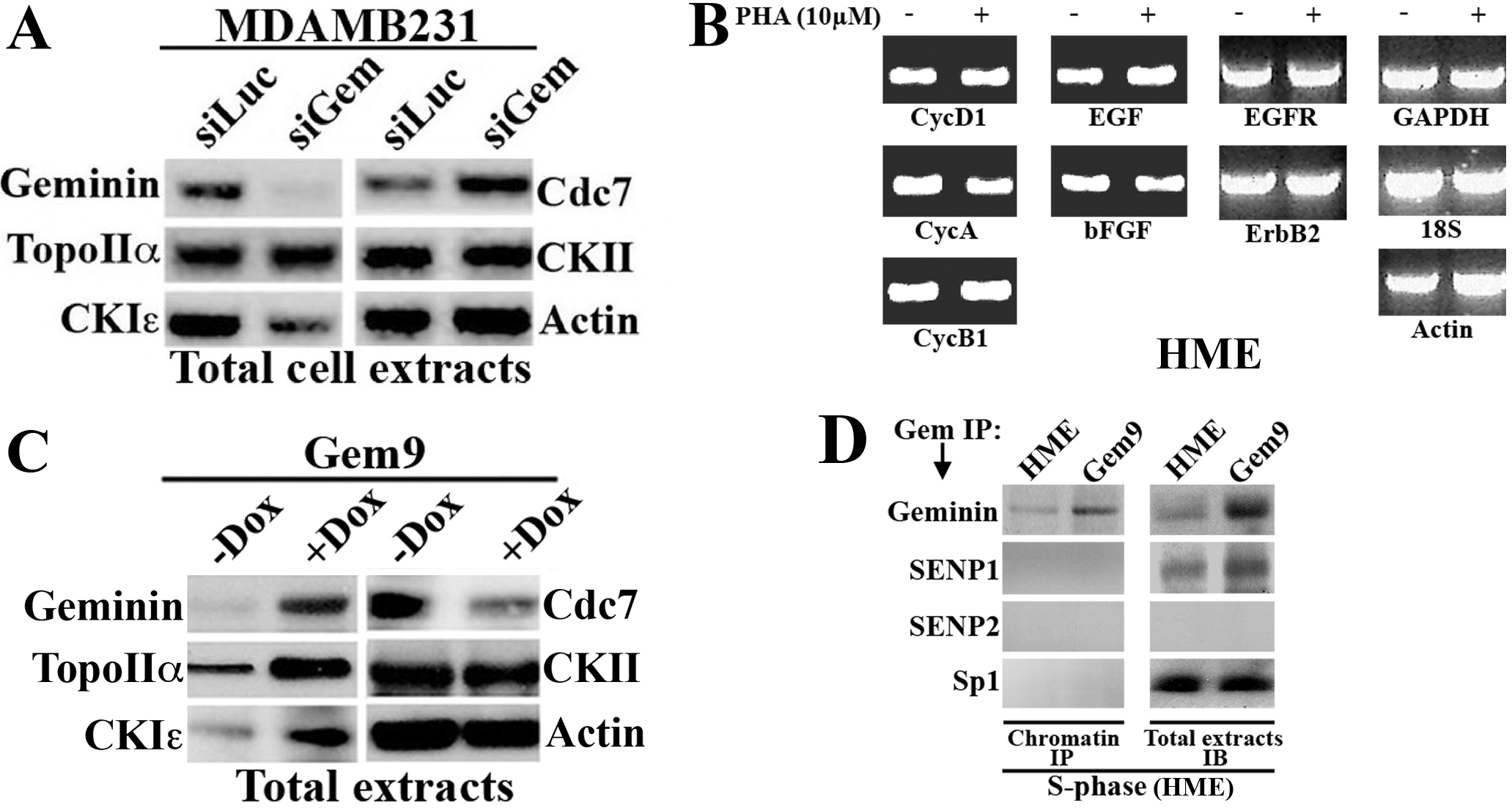

Supplement: Additional file 5 — Expression of several proteins in geminin-silenced or geminin-overexpressing cells. (A) Expression of the indicated proteins in MDAMB231 cells following geminin silencing detected using immunoblotting. (B) Expression of the indicated mRNA in control or PHA767491-treated cells detected using RT-PCR. EGF, epidermal growth factor; EGFR, epidermal growth factor receptor; GAPDH, glyceraldehyde 3-phosphate dehydrogenase; bFGF, basic fibroblast growth factor; ErB2, human epidermal growth factor receptor 2. (C) Expression of the indicated proteins in uninduced or induced (72 hours) Gem9 cells after detection using immunoblotting. Dox, doxycycline. (D) Coimmunoprecipitates of the indicated proteins with anti-geminin antibody from the chromatin of S-phase HME or induced Gem9 cells (72 hours) (left) or expression of the indicated proteins in whole cell extracts of S-phase HME or induced Gem9 cells (72 hours). IP, immunoprecipitation; IB, immunoblotting; SENP1 and SENP2, sentrin-specific protease enzymes 1 and 2. [file bcr2884-S5.TIFF]

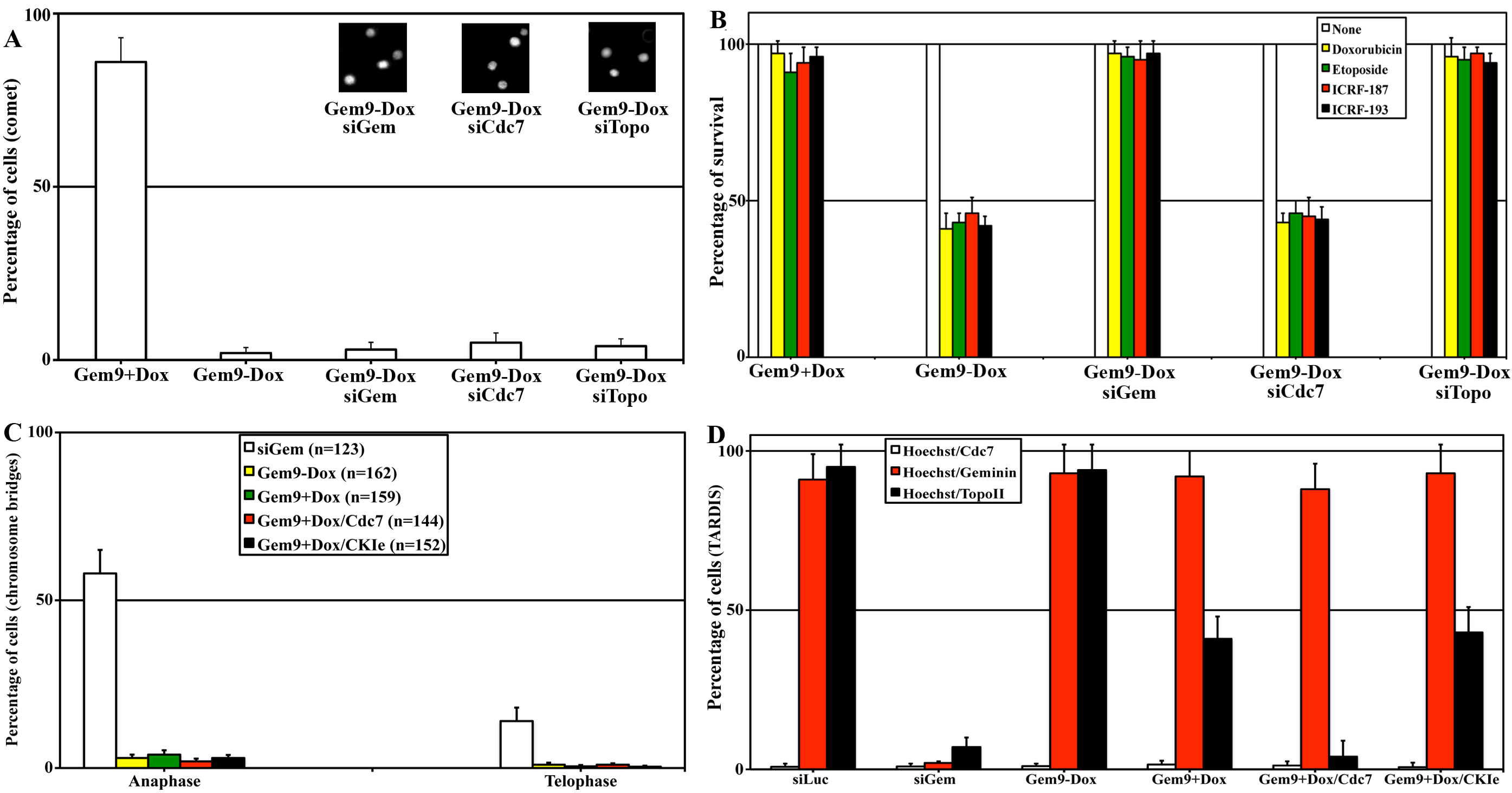

Supplement: Additional file 6 — Effects of geminin silencing or overexpression on DNA damage and drug response. (A) Comet assay comparing induced Gem9 cells to uninduced Gem9 cells following luciferase, geminin, Cdc7 or TopoIIα silencing. (B) The effect of the TopoIIα drugs doxorubicin, etoposide, ICRF187 and ICRF193 on the viability of induced Gem9 or uninduced Gem9 when luciferase, geminin, TopoIIα and Cdc7 were silenced. (C) Numbers of anaphase or telophase bridges in geminin-silenced, induced Gem9, induced Gem9 overexpressing Cdc7 or CKIε. (D) Percentage of cells stained for Hoechst 33258 blue and Cdc7, geminin or TopoIIα in luciferase- or geminin-silenced cells, uninduced or induced Gem9 cells, or induced Gem9 when Cdc7 or CKIε was overexpressed. [file bcr2884-S6.TIFF]

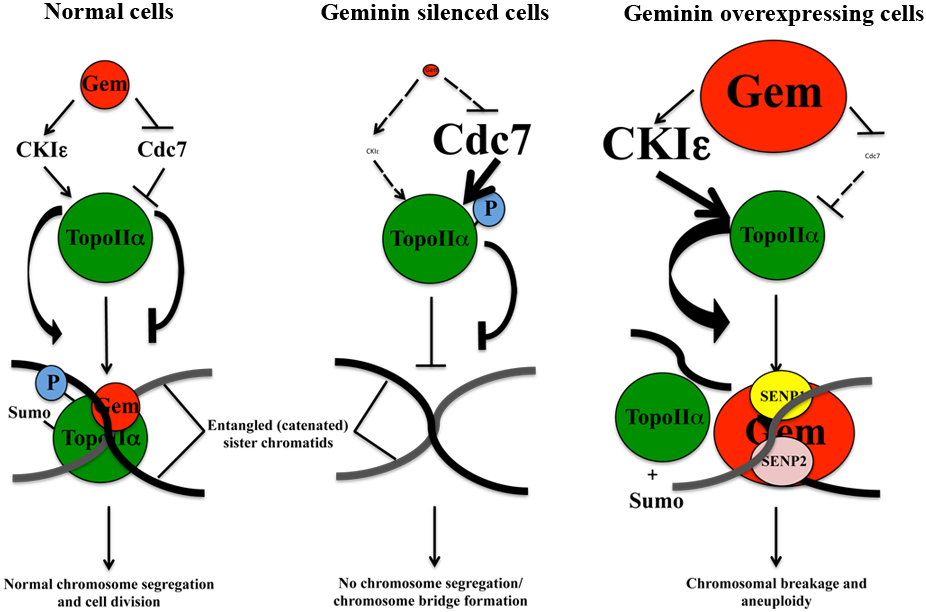

Supplement: Additional file 7 — Geminin effects on TopoIIα chromosome localization and function. Schematic model of the proposed effects of geminin in normal cells (left), geminin-silenced cells (middle) and geminin-overexpressing cells (right). [file bcr2884-S7.TIFF]
